# Phylogeny and historical demography of endemic fishes in Lake Biwa: the ancient lake as a promoter of evolution and diversification of freshwater fishes in western Japan

**DOI:** 10.1002/ece3.2070

**Published:** 2016-03-16

**Authors:** Ryoichi Tabata, Ryo Kakioka, Koji Tominaga, Takefumi Komiya, Katsutoshi Watanabe

**Affiliations:** ^1^ Graduate School of ScienceKyoto UniversityKitashirakawa‐OiwakechoSakyoKyoto606‐8502Japan; ^2^ Research Institute for Humanity and Nature457‐4 Kamigamo‐MotoyamaKita‐kuKyoto603‐8047Japan; ^3^ Kwansei Gakuin Senior High School1‐155 Uegahara‐ichibanchoNishinomiyaHyogo662‐8501Japan

**Keywords:** Ancient lake, cytochrome *b*, divergence time, historical demography, Lake Biwa, mtDNA

## Abstract

To elucidate the origins of the endemic fish of Lake Biwa, an ancient lake in Japan, and the role of the lake in the diversification of freshwater fish in western Japan, we established a molecular phylogenetic framework with an absolute time scale and inferred the historical demography of a large set of fish species in and around the lake. We used mtDNA sequences obtained from a total of 190 specimens, including 11 endemic species of Lake Biwa and their related species, for phylogenetic analyses with divergence time estimations and from a total of 2319 specimens of 42 species (including 14 endemics) occurring in the lake for population genetic analyses. Phylogenetic analysis suggested that some of the endemic species diverged from their closest relatives earlier (1.3–13.0 Ma) than the period in which the present environmental characteristics of the lake started to develop (ca. 0.4 Ma), whereas others diverged more recently (after 0.4 Ma). In contrast, historical demographic parameters suggested that almost all species, including endemic and nonendemic ones, expanded their populations after the development of the present lake environment. In phylogeographic analyses, common or very close haplotypes of some species were obtained from Lake Biwa and other regions of western Japan. The phylogenetic and historical demographic evidence suggests that there was a time lag between phylogenetic divergence and population establishment and that phenotypic adaptation of some endemic species to the limnetic environment occurred much later than the divergences of those endemic lineages. Population structure and phylogeographic patterns suggest that Lake Biwa has functioned not only as the center of adaptive evolution but also as a reservoir for fish diversity in western Japan.

## Introduction

A few dozen of ancient lakes worldwide have been in existence for hundreds of thousands or even millions of years (Cristescu et al. 2010). Ancient lakes such as the African Great Lakes, Lake Baikal, and Lake Ohrid support unique biotas, including endemic species because of their long histories as well as their various habitat assemblages, which are not seen in rivers or ponds. Owing to their historical stability, ancient lakes are thought to have functioned as glacial refugia (e.g., snails and salmonids in Lake Ohrid; Sušnik et al. 2007; Budzakoska‐Gjoreska et al. 2014). In addition, ancient lakes have been centers of adaptive evolution and speciation for freshwater organisms (Cristescu et al. 2010). Typical cases include the explosive adaptive radiation of cichlids in the African Great Lakes and cottoids in Lake Baikal, Siberia (Kontula et al. 2003; Seehausen 2006). To reveal the evolutionary histories of unique organisms and biota in ancient lakes, molecular phylogenetic and population genetic approaches have provided a fundamental basis for inferring the phylogenetic relationships and patterns of speciation of relevant species (Cristescu et al. 2010).

Lake Biwa is an ancient lake of temperate Asia, located in central Honshu Island, western Japan (35°20ʹ N, 136°10ʹ E), whose origin dates back at least 4 Ma (Yokoyama 1984; Kawabe 1989, 1994). It is the largest lake in Japan in terms of surface area (670.3 km^2^) and volume (27.5 km^3^), and it consists primarily of the northern and the southern lake basins (Kawabe 1994). The former has a large, deep pelagic zone (surface area 617.8 km^2^; mean and maximum depths 43 and 104 m, respectively) and various substrate types (i.e., rocks, pebbles, and sands), which characterize the unique environments of the present lake. By contrast, the latter is small and shallow (area 52.5 km^2^; mean and maximum depths 4 and 7 m, respectively) and is characterized by a primarily littoral environment. The lake was located farther to the southeast until about 0.4 Ma, and the present lake environment, especially in the northern basin, started to develop *c*. 0.4 Ma (the “present Lake Biwa” stage; Yokoyama 1984; Meyers et al. 1993; Kawabe 1994).

**Figure 1:**
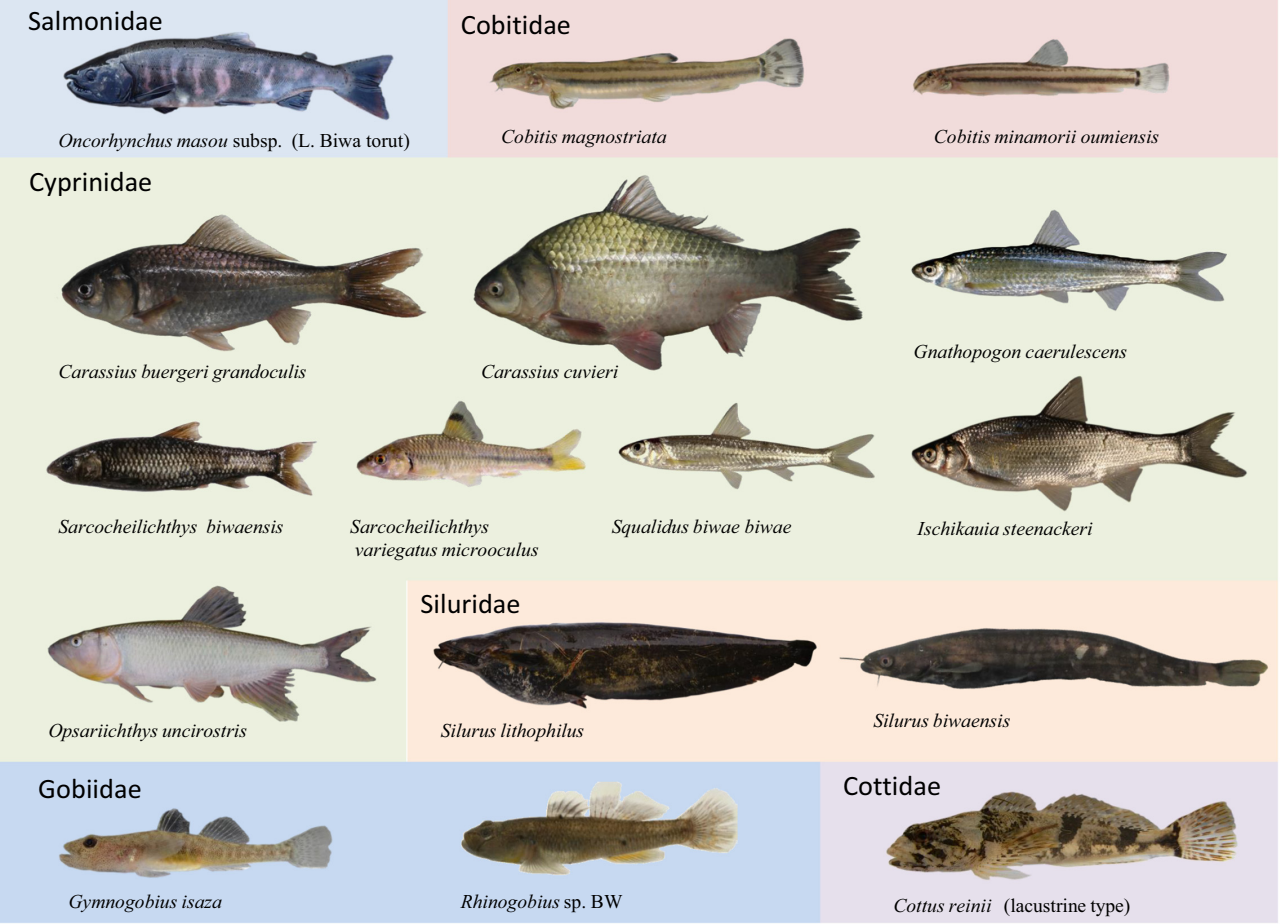
Endemic and semiendemic fishes in Lake Biwa. Note that a semiendemic species is not endemic to Lake Biwa, but its distribution is mostly restricted to Lake Biwa.

Similar to other ancient lakes, Lake Biwa harbors rich fauna and flora (>1000 species/subspecies), including ~60 endemic species/subspecies (Nishino 2003; Nishino and Hamabata 2005). In the case of fish, more than 60 species/subspecies occur in and around Lake Biwa, 16 of which are endemic or semiendemic to Lake Biwa (Fig. 1; Watanabe 2014). Thus, the lake has been recognized as biologically important because of its biodiversity and endemicity (Kawanabe 1978, 1996; Rossiter 2000). The number and proportion (<10%) of endemic forms in Lake Biwa, however, are limited compared with other ancient lakes (e.g., >600 species in Lake Tanganyika; ~1000 species in Lake Baikal; Martens 1997). This is also the case in the fish assemblage, which consists of several sets of one to a few endemics and their closely related nonendemic forms. Therefore, the fish assemblage of Lake Biwa is unique in that it lacks “species flocks,” which are species groups, such as those found in the sculpins of Lake Baikal or the cichlids of the African Great Lakes, generated via explosive adaptive radiation (Watanabe 2014).

The fish fauna of Lake Biwa has been discussed repeatedly, with a focus on the origins of endemic fishes and their adaptive evolution (Tomoda 1978; Takahashi 1989; Yuma et al. 1998). The endemic fishes of Lake Biwa are often presumed to be divided into two categories: “species that evolved in the lake” and “relict species” (Kawanabe 1978, 1996). Because most endemic fish in Lake Biwa seem to exhibit distinct adaptations to the particular environments of the lake (e.g., the extensive pelagic area, deep zone, or rocky substrate), they have been presumed to have differentiated from their ancestors through ecological adaptation (i.e., evolved via ecological speciation; Schluter 2000) after the formation of the present lake environment (*c*. 0.4 Ma; Nakajima 1987; Takahashi 1989) and have been categorized as “species that evolved in the lake” (Kawanabe 1978, 1996).

However, recent molecular phylogenetic studies have proposed that this hypothesis cannot be applied to some such endemic fish species. For example, representative endemic fishes, such as *Gnathopogon caerulescens* (a cyprinid, presumably adapted to the pelagic environment) and *Gymnogobius isaza* (a goby, presumably adapted to the deep pelagic area), were derived from their extant closest relatives much earlier (2.5–3.0 Ma) than the formation of the present environments (Harada et al. 2002; Kakioka et al. 2013; Tabata and Watanabe 2013). In contrast, other endemic cyprinids, such as *Sarcocheilichthys biwaensis* and *Sarcocheilichthys variegatus microoculus*, are, from a genetic perspective, scarcely differentiated from their sister species (Komiya et al. 2011; Komiya et al. 2014); hence, it has been inferred that they recently diverged or are in the process of differentiation. However, the phylogenetic origins and divergence times of most endemics of Lake Biwa have not been elucidated. Furthermore, the molecular markers used in previous studies differ from one another, and the data are not necessarily sufficient for divergence time estimations or population genetic analyses. A comparative study based on reliable phylogeny and divergence time estimations is required.

Aside from its endemic fauna, Lake Biwa is also unique as one of a few stable large‐scale freshwater systems in the Japanese Archipelago, which consists of islands with generally steep topographies and unstable geologies. This characteristic suggests that Lake Biwa has greatly influenced the historical development of the fish fauna of western Japan. Fossil records have revealed important information about past fish faunas and their historical changes in western Japan (Nakajima 1994; Watanabe and Takahashi 2010a). However, such information is incomplete as it has been derived from fragmentary bones, especially the pharyngeal teeth of cyprinids (Nakajima 1994; Watanabe and Takahashi 2010a). Additionally, most fossil remains in western Japan have been obtained from the Miocene and the middle Pleistocene (23–0.5 Ma; Nakajima 1994; Watanabe and Takahashi 2010a), and remains from the present Lake Biwa stage (~0.4 Ma and later) are lacking. Therefore, little information is available from fossil records about changes in the fish fauna in the present Lake Biwa and about the lake's influence on the faunas of the surrounding environments.

To understand the role of Lake Biwa in the evolution and diversification of freshwater fishes in western Japan, a molecular phylogenetic framework with an absolute time scale and historical demographic information (e.g., population bottlenecks and expansions) are essential (Avise 2000; Cristescu et al. 2010). Therefore, we first aimed to reveal the phylogenetic origins of almost all fish species endemic to Lake Biwa by using relatively long‐sequence data (~5000 bp) of various regions of mitochondrial DNA (mtDNA), basing estimated divergence times on a Bayesian approach. Based on the results, we examined whether the endemic species presumed to have evolved in the lake differentiated from their closest related species after the present Lake Biwa began to develop (~0.4 Ma). Moreover, we examined the genetic population structure and historical demographic patterns for the endemic species, together with those of many nonendemic species occurring in Lake Biwa, to clarify whether the formation of the lake fish assemblage was strongly affected by the development of the present Lake Biwa environment. Finally, based on the results of these phylogenetic and population genetic analyses, we discuss the factors that affected the evolutionary patterns of the endemic fishes of Lake Biwa and their historical effects on the fish fauna of western Japan.

## Materials and Methods

### Fish specimens

**Figure 2:**
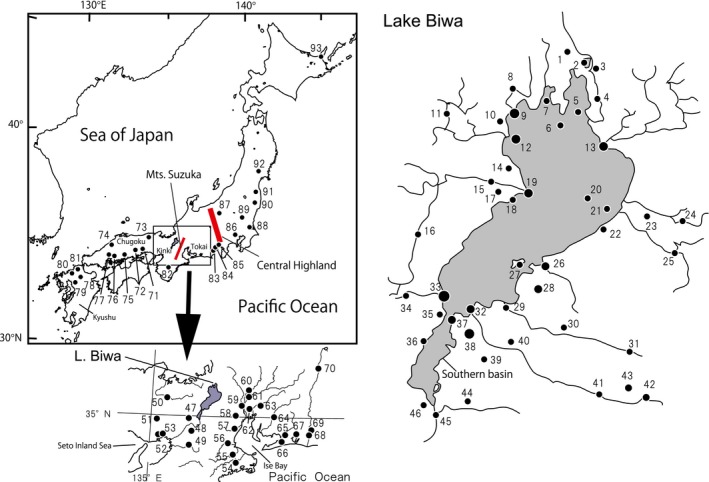
Sampling localities of specimens used for phylogenetic and population genetic analyses. For locality codes, see Tables S1 and S2.

The mtDNA sequences were obtained from a total of 190 specimens of 44 species/subspecies, including 11 of the 16 endemic/semiendemic species of Lake Biwa and their related riverine species for phylogenetic analyses, and from a total of 2319 specimens of 42 species (including 14 endemic/semiendemic species) occurring in the lake for population genetic analyses (Table 1, Fig. 2, Tables S1 and S2). Furthermore, to reveal the relationships among intraspecific clades through phylogeographic analysis, we used 497 specimens of nine species from other regions, including some data from previous studies (Table S3). Five endemic species/subspecies were not used in the phylogenetic analysis because they or their relatives exhibit polyploidy (e.g., the cobitids *Cobitis magnostriata* and *Cobitis minamorii oumiensis* (see Saitoh 2000; Kitagawa et al. 2005); and the cyprinid *Carassius buergeri grandoculis* (see Mishina et al. 2014)), known mtDNA introgression (the gobiid *Rhinogobius* sp. BW (*sensu* Akihito et al. 2013; Yamasaki et al. 2015)), or the closest relative was uncertain (the cyprinid *Ischikauia steenackeri*; Takeuchi and Hosoya 2011).

**Table 1:** List of samples used for the phylogenetic and population genetic analyses (excluding the data referred to GenBank; see Table S1)

Classification	Species^a^	n^b^	bp	k	h	*π*
**Endemic species of Lake Biwa**						
Salmonidae	*Oncorhynchus masou* subsp. (Lake Biwa trout)	2/32	677	8	0.7319	0.0016
Cobitidae	*Cobitis magnostriata*	–/36	771	6	0.7635	0.0016
	*Cobitis minamorii oumiensis*	–/46	795	25	0.9140	0.0029
Cyprinidae						
Cyprininae	*Carassius buergeri grandoculis* c	8/75	690	19	0.8541	0.0097
	*Carassius cuvieri* c	4/27	690	8	0.6781	0.0053
Gobioninae	*Gnathopogon caerulescens* e	5/54	598	15	0.7526	0.0073
	*S. variegatus microoculus* and *S. biwaensis* g	10/249	620	24	0.4626	0.0031
	*Squalidus biwae biwae*	3/99	655	30	0.9059	0.0042
Oxygastrinae	*Ischikauia steenackeri*	2/23	722	1	0.0000	0.0000
	*Opsariichthys uncirostris*	2/78	720	26	0.8923	0.0031
Siluridae	*Silurus biwaensis*	2/–	–	–	–	–
	*Silurus lithophilus*	4/24	408	7	0.6993	0.0037
Gobiidae	*Rhinogobius* sp. BW	3/43	577	22	0.9358	0.0039
	*Gymnogobius isaza* h	4/116	625	60	0.8417	0.0053
Cottidae	*Cottus reinii* (lacustrine type)	2/79	707	16	0.7063	0.0015
**Nonendemic species of Lake Biwa and outgroup species**						
Osmeridae	*Plecoglossus altivelis*	–/26	776	10	0.8400	0.0036
Salmonidae	*Oncorhynchus masou ishikawae*	2/–	–	–	–	–
	*Oncorhynchus masou masou*	2/–	–	–	–	–
Cobitidae	*Cobitis* sp. BIWAE type A	–/31	753	7	0.8301	0.0025
	*Misgurnus anguillicaudatus*	–/18	773	8	0.8301	0.0231
Cyprinidae						
Acheilognathinae	*Acheilognathus rhombeus*	–/53	749	10	0.5566	0.0012
	*Acheilognathus tabira tabira*	–/72	718	32	0.7895	0.0026
	*Tanakia lanceolata*	–/29	720	9	0.7118	0.0023
	*Tanakia limbata*	–/49	716	19	0.8759	0.0068
Cyprininae	*Carassioides acuminatus*	3/–	–	–	–	–
	*Carassius buergeri* species complex	6/–	–	–	–	–
Gobioninae	*Abbottina rivularis*	1/31	640	4	0.2430	0.0005
	*Biwia yodoensis*	1/–	–	–	–	–
	*Biwia zezera* d	4/67	651	47	0.9715	0.0055
	*Gnathopogon elongatus* e	6/98	598	13	0.6472	0.0383
	*Hemibarbus barbus*	7/56	715	3	0.6675	0.0012
	*Pseudogobio esocinus* f	8/57	689	32	0.9354	0.0202
	*Pseudorasbora parva*	9/93	705	8	0.4338	0.0017
	*Pseudorasbora pumila*	2/–	–	–	–	–
	*Pseudorasbora pugnax*	2/–	–	–	–	–
	*Pungtungia herzi*	1/–	–	–	–	–
	*Sarcocheilichthys variegatus variegatus*	10/–	–	–	–	–
	*Squalidus biwae tsuchigae*	9/–	–	–	–	–
	*Squalidus gracilis gracilis*	7/–	–	–	–	–
	*Squalidus japonicus japonicus*	4/71	629	16	0.7890	0.0024
Leuciscinae	*Rhynchocypris lagowskii steindachneri*	–/39	723	11	0.6964	0.0020
	*Rhynchocypris oxycephalus jouyi *	–/87	710	21	0.9102	0.0154
	*Tribolodon hakonensis*	–/110	712	13	0.7661	0.0045
Oxygastrinae	*Hemigrammocypris rasborella*	8/–	–	–	–	–
	*Nipponocypris sieboldii*	1/72	704	22	0.9354	0.0058
	*Nipponocypris temminckii*	2/42	707	3	0.0941	0.0003
	*Zacco platypus*	6/62	674	37	0.9688	0.0064
Amblycipitidae	*Liobagrus reinii*	–/16	743	7	0.6917	0.0016
Bagridae	*Pseudobagrus nudiceps*	–/26	662	4	0.5908	0.0010
Siluridae	*Silurus asotus*	18/39	409	9	0.7746	0.0096
Gobiidae	*Rhinogobius flumineus*	–/22	577	4	0.5931	0.0083
	*Rhinogobius* sp. OR	–/30	577	15	0.8322	0.0025
	*Gymnogobius breunigii*	1/–	–	–	–	–
	*Gymnogobius* sp. 1 (Musashino‐juzukakehaze)	2/–	–	–	–	–
	*Gymnogobius opperiens*	3/–	–	–	–	–
	*Gymnogobius petschiliensis*	4/–	–	–	–	–
	*Gymnogobius urotaenia* i	7/43	625	7	0.3012	0.0006
Cottidae	*Cottus pollux* ME	2/–	–	–	–	–
	*Cottus pollux* LE	6/11	707	5	0.7091	0.0012
	*Cottus reinii* (amphidromous type)	6/–	–	–	–	–

n, number of specimens; k, number of haplotypes; bp, base pairs of the sequence used for population genetic analyses; h, haplotype diversity; *π*, nucleotide diversity.

a The endemic and semiendemic fish species of Lake Biwa are divided based on Watanabe (2014).

b Number of specimens used for phylogenetic/population genetic analyses.

c From Mishina et al. (2014).

d From Watanabe et al. (2010b, b).

e From Kakioka et al. (2013).

f 44 specimens from Tominaga et al. (2009, 2015).

g From Komiya et al. (2011, 2014).

h Three specimens from Harada et al. (2002); 113 specimens from Tabata and Watanabe (2013).

i From Tabata and Watanabe (2013)

### mtDNA sequencing

Total genomic DNA was isolated from fin clips preserved in 100% ethanol using a Genomic DNA Purification kit (Promega, Tokyo, Japan). The mitochondrial nucleotide sequences of the 16S ribosomal RNA (16S rRNA), cytochrome c oxidase I (CO1), NADH dehydrogenase subunit 5 (ND5), cytochrome *b* (cyt *b*), and control region (CR) genes were chosen as molecular markers to infer phylogenetic relationships and divergence times because large amounts of data and information have been accumulated for these genes with respect to molecular evolution in fish (e.g., Briolay et al. 1998; Zardoya and Doadrio 1999; Watanabe and Takahashi 2010). These five different regions of mtDNA have different evolutionary rates (Meyer 1994; Avise 2000; Kuo et al. 2003); hence, we expected these genes to enable a comprehensive estimation of divergence times over relatively short or long time scales. In population genetic analyses, we used the cyt *b* gene (or the CR gene, see below) because of its higher substitution rate (see “Results”).

The primers described in Table S4 were used for polymerase chain reaction (PCR) amplification of the five mitochondrial regions. The PCR conditions consisted of 30 cycles of denaturation (94°C, 15 s), annealing (48°C to 55°C for each region, 15 s), and extension (72°C, 30 s) in a PC808 thermal cycler (Astec, Fukuoka, Japan). After purifying the PCR products by treatment with ExoSAP‐IT (USB Corp., Cleveland, OH, USA) at 37°C, they were sequenced using an ABI 3130xl DNA sequencer (Applied Biosystems, Foster City, CA, USA) with amplification primers using the BigDye Terminator Cycle Sequencing FS Ready Reaction kit ver. 3.1 (Applied Biosystems).

The sequences of the 5′‐halves of the 16S rRNA gene (~1200 bp), CO1 gene (~600 bp), and ND5 gene (~1000 bp); the nearly complete cyt *b* gene (~1100 bp; or 580–750 bp for population genetic analyses); and the nearly complete (~900 bp) or 5′‐half of the CR gene (450 bp for silurids) were deposited in DDBJ/EMBL/GenBank (accession nos. LC097321–098734; Tables S1–S3). The haplotype frequencies for populations are available from GEDIMAP (http://gedimap.zool.kyoto-u.ac.jp/; Watanabe et al. 2010a) under accession numbers P2000–2195. For population genetic analyses, the sequence data of some species were drawn from Harada et al. (2002), Tominaga et al. (2009, 2015), Watanabe et al. (2010b), Komiya et al. (2011), Kakioka et al. (2013), Tabata and Watanabe (2013), and Mishina et al. (2014).

### Phylogenetic reconstruction and estimation of divergence time

For phylogenetic reconstruction and estimation of the divergence times of endemic fishes in Lake Biwa, we used the mtDNA sequence data of 11 endemic species/subspecies, their closely related species, and outgroups, including some data from previous studies. Datasets were created for (1) Salmonidae, (2) Cyprininae (Cyprinidae), (3) Gobioninae (Cyprinidae), (4) Oxygastrinae (Cyprinidae), (5) Siluridae, (6) Gobiidae, and (7) Cottidae (Table S1).

The sequences of the five mitochondrial regions were aligned using the Muscle (Edgar 2004) and Mafft (Katoh et al. 2002) software. These alignments were automatically trimmed with trimAl (Capella‐Gutiérrez et al. 2009) to remove poorly aligned regions with gaps in more than 20% of the sequences or with a similarity score lower than 0.001, unless this removed more than 40% of the columns in the original alignment before the sequences were concatenated. For each dataset, the Bayesian approach was applied to estimate the phylogenetic trees, divergence times, and molecular evolutionary rates for the lineages of the endemic forms, with the fittest models selected by the Bayesian information criterion (BIC) in jModelTest v2.1.1 (Table S5; Darriba et al. 2012) and Yule (speciation) tree prior using BEAST v1.6.2 (Drummond and Rambaut 2007). We adopted the random local clock model, which assumes one or more independent rates on different branches (Drummond and Suchard 2010). To estimate the age of divergence between lineages, we imposed some a priori constrains on the node ages, mostly from geological events (such as upheavals of mountains) that divided regional populations of freshwater fish (Table 2). No calibration point was available for *Gymnogobius isaza* because of the lack of fossil data and the unclear geographic population structure of its closely related species. Thus, the previously estimated evolutionary rate of the *G. isaza* cyt *b* gene (Tabata and Watanabe 2013) was adopted (Table 2).

**Table 2:** List of constrains used for the estimation of divergence times of endemic fish species in Lake Biwa

Node	Event	Code^a^	Mya	Prior distribution^b^	Reference
**Salmonidae**					
the basal of *Oncorhynchus* species	the fossil of *Oncorhunchus*	C5	12–23	LN; mean = 18 Mya, log (SD) = 0.2, offset = 0	Behnke (2002), Eiting and Smith (2007), Wilson and Turner (2009)
*Oncorhynchus mykiss* – *O. clarki*	the fossil of *O. mykiss*	C6	1.0–3.0	LN; mean = 2.4 Mya, log (SD) = 0.4, offset = 0	Behnke (2002), Wilson and Turner (2009)
**Cyprinidae**					
**Cyprininae**					
*Carassius carassius – C. buergeri (auratus)*	the fossil of *C. auratus*	C3	3.5–4.0	LN; mean = 3.8 Mya, log (SD) = 0.06, offset = 0	Gao et al. (2012)
**Gobioninae**					
Ise – L. Biwa population (*Biwia zezera*)	the uplift of Mts. Suzuka	C1	1.0–1.5	LN; mean = 1.25 Mya, log (SD) = 0.09, offset = 0	Watanabe et al. (2010b)
Node of East (*Gnathopogon elongatus*)	the formation of Ina Valley	C2	0.8–	IG; the shape parameter =2,scale = 3,offset = 0	Kakioka et al. (2013)
West – East clade (*Pseudogobio esocinus*; G1)	the uplift of Mts. Suzuka	C1	1.0–1.5	LN; mean = 1.25 Mya, log (SD) = 0.09, offset = 0	Tominaga et al. (2009, 2015)
West – East clade (*Pseudogobio esocinus*; G2)	the uplift of Mts. Suzuka	C1	1.0–1.5	LN; mean = 1.25 Mya, log (SD) = 0.09, offset = 0	Tominaga et al. (2009, 2015)
West – East clade of *Pseudorasbora parva*	the uplift of Mts. Suzuka	C1	1.0–1.5	LN; mean = 1.25 Mya, log (SD) = 0.09, offset = 0	
*Pseudorasbora pumila* – *P. pugnax*	the uplift of Central Highland	C4	2.0–5.0	LN; mean = 3.5 Mya, log (SD) = 0.29, offset = 0	
Ise – Western Japan clade (*Sarcocheilichthys* fish)	the uplift of Mts. Suzuka	C1	1.0–1.5	LN; mean = 1.25 Mya, log (SD) = 0.09, offset = 0	Komiya et al. (2014)
West – East clade (*Squalidus gracilis gracilis*)	the uplift of Mts. Suzuka	C1	1.0–1.5	LN; mean = 1.25 Mya, log (SD) = 0.09, offset = 0	
**Oxygastrinae**					
West – East clade (*Hemigrammocypris rasborella*)	the uplift of Mts. Suzuka	C1	1.0–1.5	LN; mean = 1.25 Mya, log (SD) = 0.09, offset = 0	Watanabe et al. (2014)
West – East clade (*Zacco platypus*)	the uplift of Mts. Suzuka	C1	1.0–1.5	LN; mean = 1.25 Mya, log (SD) = 0.09, offset = 0	
**Siluridae**					
*Silurus asotus* (Eastern) *– S. lithophilus*	the uplift of Mts. Suzuka	C1	1.0–1.5	LN; mean = 1.25 Mya, log (SD) = 0.09, offset = 0	
**Gobiidae**					
*Gymnogobius castaneus–taranetzi* species complex	the inflow of Tsushima Current	–	2.5–4.5	LN; mean = 3.55 Mya, log (SD) = 0.17, offset = 0	Tabata and Watanabe (2013)
**Cottidae**					
West – East clade (*Cottus pollux* LE)	the uplift of Mts. Suzuka	C1	1.0–1.5	LN; mean = 1.25 Mya, log (SD) = 0.09, offset = 0	

a For codes, see Fig. 3 or Fig. S1.

b LN, a lognormal distribution; IG, an inverse‐gamma distribution.

All other model parameters used default priors. For each Markov chain Monte Carlo (MCMC) analysis, we performed two independent runs of 100 million generations, sampling every 1000th generation and removing the initial 10% of samples as burn‐in. The convergence of the chains to the stationary distribution was confirmed by inspection of MCMC samples using Tracer v1.5 (Rambaut and Drummond 2009). These two runs were combined, and the consensus tree was calculated using TreeAnnotator v1.6.2 in the BEAST package and visualized using FigTree v1.3.1 (Rambaut 2009).

### Population genetic and historical demographic analyses

For population genetic analyses, we used the cyt *b* sequences of a total of 2319 specimens from 42 species (11–249 specimens/species) occurring in Lake Biwa, including 11 endemic forms (Table S2). Because *Silurus lithophilus* exhibited no genetic diversity in the cyt *b* gene, we used the CR gene, which of silurids showed a faster evolutionary rate and genetic polymorphism (see “Results”), for analyses of *S. lithophilus* and *Silurus asotus*. We treated *Sarcocheilichthys biwaensis* and *S. variegates microoculus* as the species complex “*Sarcocheilichthys* spp.” in the population analysis because no genetic difference was detected between them in the mtDNA (cyt *b*) and microsatellite markers (Komiya et al. 2011).

The genetic diversity of endemic fish was evaluated based on haplotype diversity (*h*) and nucleotide diversity (*π*) using the Arlequin 3.5 software (Excoffier and Lischer 2010). The relationships among mtDNA haplotypes of each species were estimated as a statistical parsimony network using the TCS 1.2.1 software (Clement et al. 2000). When we found distinctive multiple intraspecific mitochondrial clades in Lake Biwa, we treated those clades both together and separately in the following analyses, and added the data of specimens from other regions to examine the origins of the clades (Table S3).

To evaluate whether sequences had evolved under strict neutrality, Fu's *F*_*S*_ (Fu 1997) was calculated using Arlequin. Fu (1997) noticed that the 2% percentile of the distribution corresponded to the 5% cutoff value. Therefore, when the statistic of Fu's *F*_*S*_ became negative at the significance level of 2%, population expansion or selection was suggested. To examine the possible occurrence of a historical demographic expansion of each species, we performed mismatch distribution analysis (Rogers and Harpending 1992), which was implemented in Arlequin. The fitness of the observed population data to a model of sudden population expansion (Rogers and Harpending 1992) was tested by calculating the sum of the squared deviation (SSD) and raggedness (rg) statistics (Harpending 1994). For each statistic, the sudden expansion model was rejected when *P *< 0.05 (Excoffier and Lischer 2010). The parameter *τ* is related to the time of population expansion by the formula *τ *= 2*Tu*, where *T* measures time in years when *u* is the mutation rate per nucleotide per year, multiplied by the sequence length (Slatkin and Hudson 1991). We estimated the time of population expansion with the rate (*u*) obtained from the Bayesian phylogenetic analysis based on the random local clock model mentioned above or the rate reported in a previous study (Zardoya and Doadrio 1999; Kawamura et al. 2014; see Table S5).

The demographic history of the fishes in Lake Biwa, including endemics, was also examined by Bayesian skyline plot (BSP) analysis (Drummond et al. 2005), implemented in BEAST. We performed runs with a MCMC chain length of 50 million generations, sampling every 1000th generation and removing the initial 10% of samples as burn‐in. The substitution model used was the model selected by BIC in jModelTest, and the time to expansion was estimated using the mutation rate obtained in the above phylogenetic analysis. The BSP result with the stepwise (constant) model was summarized using Tracer. When we found two or more distinct intraspecific mitochondrial clades in the TCS analysis, we carried out mismatch distribution and BSP analyses for each intraspecific clade separately.

## Results

### Divergence time estimation based on mtDNA genes

The evolutionary rates (nucleotide substitutions per million years per lineage) of the respective genes at the endemic species lineages were estimated to be 0.18–0.41% (16S rRNA), 0.37–1.00% (CO1), 0.44–1.82% (ND5), 0.53–1.89% (cyt *b*), and 0.27–1.57% (CR) (Table S5). In most species, the cyt *b* or ND5 genes showed the highest average molecular evolution rate, whereas the 16S rRNA gene exhibited the lowest rate. Exceptionally, the rate of CR was the highest in the *Silurus* species (Table S5).

The degree of genetic divergence between the endemic species in Lake Biwa and their closely related species varied highly between species. Some species showed relatively large genetic distances (uncorrected *P*): 3.9% (between *Opsariichthys uncirostris* and *Opsariichthys bidens*) to 6.5% (between *Silurus* species). Other species, such as *Squalidus* spp. and *Sarcocheilichthys* spp., exhibited small genetic distances (<1%) (Table 3). These reflected the wide range of estimated divergence times of the endemic lineages (Table 3; Figs. 3 and 4).

**Table 3:** Divergence times of endemic fish species in Lake Biwa

Node	Code	*P*‐ dis.	tMRCA (Myr)	95% HPD
**Salmonidae**				
*Oncorhynchus masou* subsp. (Lake Biwa trout) *‐ O. masou* subspecies	a	0.007	0.52	0.29–0.76
**Cyprinidae**				
*Carassius cuvieri ‐ Carassius* species (China & Japan)	b	0.055	2.64	2.30–3.00
*Gnathopogon caerulescens ‐ G. elongatus* (West)	c	0.026	2.39	1.81–2.96
*S. v. variegatus* ‐ *S. biwaensis ‐ S. v. microoculus*	d	<0.005	0.07–0.52	0.02–0.67
*Squalidus biwae biwae ‐ S. b. tsuchigae* (Setouchi & around L. Biwa)	e	<0.006	0.18–0.56	0.10–0.72
*Opsariichthys unicirostris* ‐ *O. bidens*	f	0.039	2.99	2.01–3.96
**Siluridae**				
*Silurus lithophilus ‐ S. asotus* (Eastern)	g^a^	0.008	1.24	1.02–1.45
*S. lithophilus + S. asotus* (Eastern) *‐ S. asotus* (Western, including Lake Biwa)	h	0.052	9.67	6.34–13.3
*S. lithophilus + S. asotus ‐ S. biwaensis*	i	0.065	13.0	8.56–17.9
**Gobiidae**				
*Gymnogobius isaza ‐ G. urotaenia + G. petschiliensis*	j	0.062	2.25	1.82–2.65
**Cottidae**				
*Cottus reinii ‐ C. reinii* (amphidromous type; Ise Bay)	k	0.011	1.25	1.05–1.46

For node codes, see Fig. 3. *P‐dis*. uncorrected *P*‐distance.

a Calibration point.

**Figure 3:**
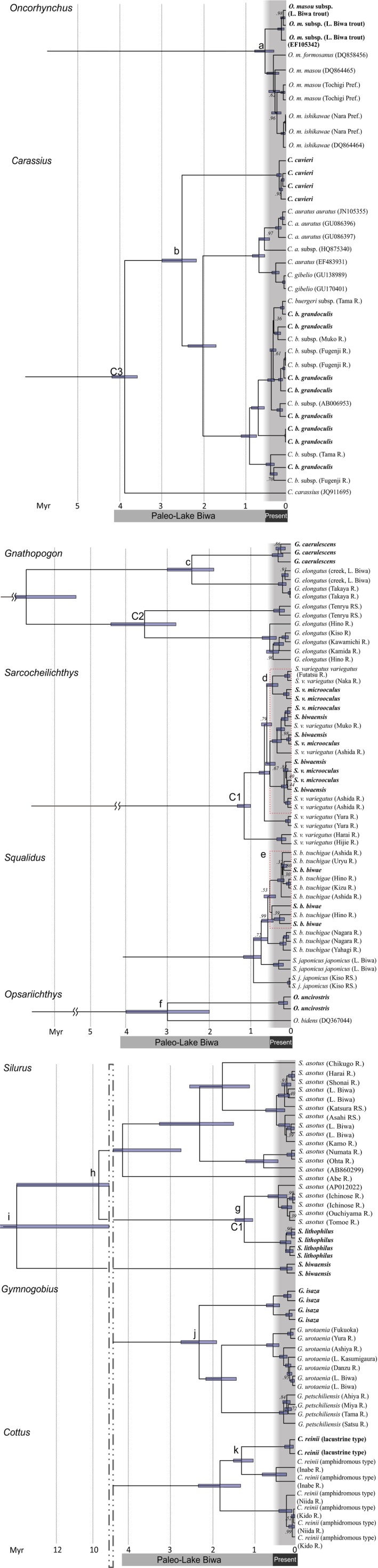
Bayesian phylogenetic trees of each fish group including species/subspecies endemic to Lake Biwa based on mtDNA 16S rRNA, CO1, ND5, cyt *b*, and CR gene sequences. Outgroups are not shown. The trees were dated by the random local clock model with node age constraints (C1–C6; see Table 2 and Fig. S1). All nodes are supported by a Bayesian posterior probability of 1.0, except for those denoted by *numbers*. *Horizontal bars at nodes* show credible intervals as 95% highest posterior density. For codes of nodes (a–o), see Table 3. For results including outgroups, see Fig. S1.

**Figure 4:**
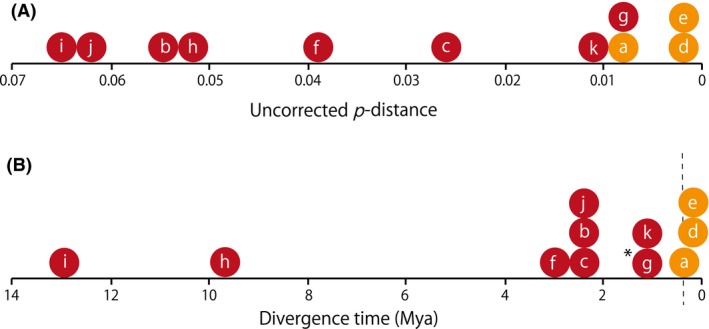
(A) Distribution of uncorrected *P* distances and (B) timing of divergence between endemic fishes and their relatives in Lake Biwa. *a*,* Oncorhynchus masou* subsp. “Lake Biwa trout” (Salmonidae); *b*,* Carassius cuvieri* (Cyprinidae); *c*,* Gnathopogon caerulescens* (Cyprinidae); *d*,* Sarcocheilichthys biwaensis* and *S. variegatus microoculus* (Cyprinidae); *e*,* Squalidus biwae biwae* (Cyprinidae); *f*,* Opsariichthys uncirostris* (Cyprinidae); *g*,* Silurus lithophilus* (Siluridae; vs. one sister cryptic clade of *S. asotus*); *h*,* S. lithophilus* (Siluridae; vs. all clades of *S. asotus*); *i*,* Silurus biwaensis* (Siluridae); *j*,* Gymnogobius isaza* (Gobiidae); *k*,* Cottus reinii* (lacustrine type) (Cottoidae).

Dividing the endemic species based on a divergence time of ~0.4 Myr (0.3–0.5 Myr), when the present Lake Biwa started to develop, the estimation showed that seven forms diverged prior to the development of the present lake, whereas the remaining four diverged more recently. Of the former group, the divergence time of *Silurus biwaensis* from the common ancestor of *S. asotus* and *S. lithophilus* was the oldest (13 Myr), and these species formed a monophyletic group that includes Eurasian continental species. The divergence time of *S. lithophilus* from *S. asotus* in western Japan was the second oldest (9 Myr), but the former did not show a sister relationship to the latter; however, it did show such a relationship to the cryptic clade of *S. asotus* occurring in the eastern area (1.24 Myr, calibration point C1). The divergence times of *Opsariichthys uncirostris* (from *O. bidens*), *Gnathopogon caerulescens* (from the west clade of *G. elongatus*), *Carassius cuvieri* (from *Carassius* species in China and Japan), and *Gymnogobius isaza* (from the common ancestor of *G. urotaenia* and *G. petschiliensis*) were estimated at ~2–3 Myr The divergence time of *Cottus reinii* lacustrine type (from the Ise Bay area population of *Cottus reinii* amphidromous type) was estimated at 1.3 Myr.

On the other hand, the other four endemic forms, *Squalidus biwae biwae*,* Sarcocheilichthys biwaensis*,* S. variegates microoculus*, and *Oncorhynchus masou* subsp. “Lake Biwa trout,” were estimated to have diverged from their closely related species more recently (Table 3, Figs. 3 and 4). Except for the Lake Biwa trout, the other three forms showed no distinct genetic divergence in their mtDNA from their closely related species.

### Population genetic and historical demographic analyses

**Figure 5:**
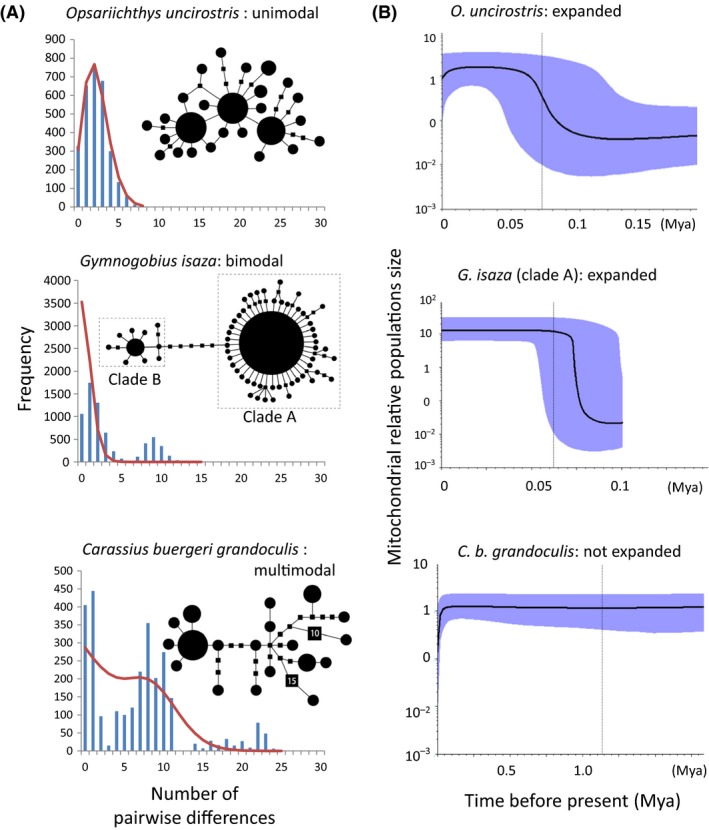
(A) Mismatch distributions and statistical parsimony networks of mtDNA cytochrome *b* haplotypes of fishes in Lake Biwa. In mismatch distribution results, the *bars* of the histogram represent the frequencies of the observed pairwise differences. The *solid lines* represent the expected distributions under a sudden expansion model. In haplotype networks, the *sizes of the circles* are proportional to the numbers of individuals. A *square* indicates a missing haplotype. The number of missing haplotypes (≥2) is indicated *in the squares*. The *branches* indicate one substitution. (B) The typical results of Bayesian skyline plots for representative endemic species. The *central bold line* represents the median value for the relative effective population size, and the *solid area* denotes the 95% upper and lower credible limits. For detailed statistics and results of other species, see Table 4 and Table S6 and Fig. S2.

The endemic species showed a wide range of the haplotype diversity (*h*; 0.000–0.9358, average ± SD = 0.724 ± 0.244 in cyt *b* or CR; see “Materials and Methods”). Among 14 endemic fishes, 10 had *h* values higher than 0.7. By contrast, *Ischikauia steenackeri* had no genetic variation in its mtDNA cyt *b* gene (Table 1). The haplotype networks of the respective fishes in Lake Biwa, including endemics, showed various patterns including star‐like, bush‐like, and dumbbell‐like patterns (Figs. 5 and S2). The cyt *b* haplotypes of *Sarcocheilichthys* spp. and *Gymnogobius isaza* were clearly divided into three and two clades, respectively. Hereafter, the clades of *Sarcocheilichthys* spp. are treated as “clade A” (main clade), “clade B,” and “clade C;” and those of *Gymnogobius isaza* are treated as “clade A” (main clade) and “clade B” (Figs. 5 and S2).

**Figure 6:**
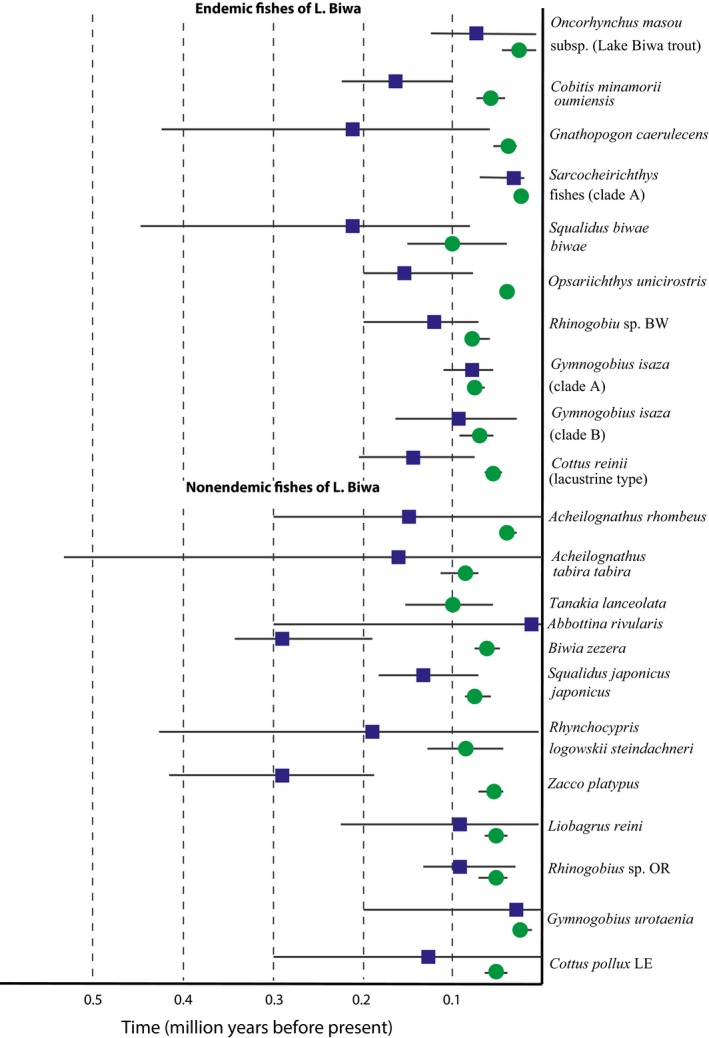
Timing of population expansion in the populations of endemic and nonendemic fishes in Lake Biwa. The *squares* represent the time based on the mismatch distribution analysis. The *circles* represent the time estimated with the Bayesian skyline plot analysis. *Bars* denote the 95% upper and lower credible limits.

Fu's *F*_*S*_ suggested that the null hypothesis of neutrality was rejected in nine of 14 endemic forms (i.e., *Oncorhynchus masou* subsp., *Cobitis minamorii oumiensis*,* Opsariichthys uncirostris*,* Squalidus biwae biwae*,* Gnathopogon caerulescens*,* Sarcocheilichthys* spp., *Rhinogobius* sp. BW, *Gymnogobius isaza*, and *Cottus reinii* (lacustrine type)) and three intraspecific clades (i.e., clade A of *Sarcocheilichthys* spp. and clades A and B of *G. isaza*) (Table 4). In the mismatch distribution analysis, these endemic species, with the exception of *Sarcocheilichthys* spp. and *G. isaza*, fit the model of sudden expansion and exhibited unimodal distributions (Table 4, Figs. 5 and S2). The mismatch distributions for *Sarcocheilichthys* spp., *Carassius buergeri grandoculis*,* C. cuvieri*, and *G. isaza* were multimodal (Figs. 5 and S2). Using the evolutionary rate estimated by the above phylogenetic analysis, the population expansion times of the respective endemic forms and intraspecific clades were estimated at 0.03–0.22 Ma (average ± SD = 0.13 ± 0.06; Table 4, Fig. 6) (see Table S5).

**Table 4:** Results of mismatch distribution analyses and neutrality tests (see Table S6 for more detailed data)

Species	*n*	Fu F_S_	*P*_*ssd*_	*P*_*rg*_	*τ*	*T* (10^4 ^yrs)	*BSP*	*tMRCA* (10^4 ^yrs)
**Endemic species of Lake Biwa**								
Salmonidae								
*Oncorhynchus masou* subsp. (L. Biwa torut)	32	−3.55**	0.184	0.070	1.17	7.4	exp.	7.4
Cobitidae								
*Cobitis magnostriata*	36	−0.82	0.230	0.222	–	–	stab.	17.4
*Cobitis minamorii oumiensis*	46	−23.69**	0.381	0.404	2.46	16.6	exp.	30.8
Cyprinidae								
Cyprininae								
*Carassius buergeri grandoculis*	75	−0.12	0.355	0.401	–	–	stab.	188.3
*Carassius cuvieri*	27	0.50	0.093	0.055	–	–	stab.	54.9
Gobioninae								
*Gnathopogon caerulescens*	54	−11.46**	0.126	0.261	2.43	21.8	exp.	20.0
*Sarcocheilichthys* species	249	−11.44**	0.345	0.732	–a	–a	–	69.1
(clade A)	222	28.15**	0.281	0.532	0.36	3.1	exp.	3.4
(clade B)	14	−0.04	0.498	0.645	–	–	stab.	5.2
(clade C)	13	0.44	0.154	0.148	–	–	stab.	6.1
*Squalidus biwae biwae*	95	−20.58**	0.865	0.844	2.59	21.3	exp.	32.9
Oxygastrinae								
*Ischikauia steenackeri*	23	—	—	–	–	–	–	–
*Opsariichthys uncirostris*	77	−20.10**	0.500	0.584	2.31	15.3	exp.	9.4
Siluridae								
*Silurus lithophilus*	24	−1.36	0.357	0.441	–	–	stab.	18.5
Gobiidae								
*Rhinogobius* sp. BW	43	−8.39**	0.515	0.305	2.06	12.2	exp.	19.5
*Gymnogobius isaza*	116	−26.22**	<0.001**	1	–	–	–	40.4
(clade A)	100	−28.09**	0.854	0.449	1.5	8.2	exp.	10.0
(clade B)	16	−6.81**	0.585	0.344	1.72	9.2	exp.	10.6
Cottidae								
*Cottus reinii* (lacustrine type)	79	−11.87**	0.229	0.095	1.14	13.9	exp.	14.6
**Nonendemic species of Lake Biwa**								
Osmeridae								
*Plecoglossus altivelis*	26	−2.12	0.678	0.868	**–**	**–**	stab.	28.6
Cobitidae								
*Cobitis* sp. BIWAE type A	31	−0.59	0.902	0.870	**–**	**–**	stab.	18.4
*Misgurnus anguillicaudatus*	16^b^	0.03*	0.375	0.272	**–**	**–**	stab.	10.2
Cyprinidae								
Acheilognathinae								
*Acheilognathus rhombeus*	53	−5.94**	0.839	0.857	0.96	12.3	exp.	8.52
*Acheilognathus tabira tabira*	72	−27.41**	0.939	0.956	1.02	13.7	exp.	10.2
*Tanakia lanceolata*	29	−3.07*	0.090	0.098	–	–	exp.	55.2
*Tanakia limbata*	49	−4.28	0.024*	0.431	**–**	**–**	stab.	82.7
Gobioninae								
*Abbottina rivularis*	31	−2.32**	0.575	0.569	0.08	0.7	**–**	0.1
*Biwia zezera*	67	−26.11**	0.885	0.606	3.44	28.4	exp.	34.7
*Gnathopogon elongatus*	98	23.63	0.066	0.155	**–**	**–**	stab.	646
*Hemibarbus barbus*	56	1.72	0.042	0.007**	**–**	**–**	stab.	7.6
*Pseudogobio esocinus*	57	−8.09*	0.345	0.348	**–**	**–**	stab.	78.6
*Pseudorasbora parva*	93	−0.12	0.502	0.604	**–**	**–**	stab.	75.2
*Squalidus japonicus japonicus*	71	−10.14**	0.291	0.149	1.42	12.1	exp.	16.2
Leuciscinae								
*Rhynchocypris lagowskii steindachneri*	39	−5.21**	0.768	0.940	2.16	19.7	exp.	16.8
*Rhynchocypris oxycephalus jouyi *	87	1.74	0.032*	0.100	**–**	**–**	stab.	82.4
*Tribolodon hakonensis*	110	−0.41	0.111	0.166	**–**	**–**	stab.	45.6
Oxygastrinae								
*Nipponocypris sieboldii*	72	−6.29**	0.680	0.934	**–** a	**–** a	stab.	72.2
*Nipponocypris temminckii*	42	−1.54*	0.151	0.779	**–**	**–**	–	0.1
*Zacco platypus*	62	−25.79**	0.963	0.881	4.22	29.8	exp.	18.6
Amblycipitidae								
*Liobagrus reinii*	16	−3.53**	0.764	0.876	1.32	9.6	exp.	13.8
Bagridae								
*Pseudobagrus nudiceps*	26	−0.65	0.049*	0.047*	**–**	**–**	exp.	7.1
Siluridae								
*Silurus asotus*	39	0.874	0.277	0.251	**–**	**–**	stab.	68.2
Gobiidae								
*Rhinogobius flumineus*	22	5.74	0.065	0.005**	**–**	**–**	stab.	212
*Rhinogobius* sp. OR	30	−13.19**	0.223	0.091	1.52	9	exp.	16.6
*Gymnogobius urotaenia*	43	−6.32**	0.632	0.585	0.48	2.6	exp.	6.8
Cottidae								
*Cottus pollux* LE	11	−2.42**	0.183	0.168	1.11	13.5	exp.	14.1

*Fu Fs*, values of Fu's neutrality test; *BSP*, Bayesian skyline plot analysis; *P*_*ssd*_ SSD *P* values; *P*_*rg*_ raggedness index *P‐*values of mismatch distribution analysis; *τ*, time parameter; *T*, estimated expansion time using each molecular clock; *tMRCA*, time to most recent common ancestor.

*P <* *0.05, **P <* *0.02. exp., a population expanded in the past; stab., a stable population size over time

Following Fu (1997) and Excoffier and Lischer (2010), Fu's *F*_S_ test was considered significant when the *P*‐value is < 0.02.

^a^ *τ* and *T* were not estimated because the population were including some mtDNA clusters and the mismatch distribution result were multimodal, although the *P*‐value of Fu's *F*_S_ was <0.02.

^b^Two individuals with haplotypes of continental clades were excluded because they were inferred to have been introduced artificially.

On the other hand, in 12 of 27 nonendemic forms, Fu's *F*_*S*_ suggested that the null hypothesis of neutrality was rejected (*P* < 0.02). The mismatch distribution of those 12 forms fit the model of sudden expansion and, with the exception of *Nipponocypris sieboldii*, which showed a clearly bimodal distribution, all exhibited a unimodal distribution (Table 4, Figs. 5 and S2). The population expansion times of these 11 nonendemic forms were estimated at 0.01–0.35 Ma (average ± SD = 0.14 ± 0.09; Table 4, Fig. 6). For the other 15 nonendemic forms, the null hypothesis of neutrality was not rejected by Fu's *F*_*S*_ (*P* > 0.02) or the model of sudden expansion was not supported by the mismatch distribution analysis (*P* < 0.05).

The BSP analysis also revealed the population expansion pattern in most of the endemic forms, which began ca. 0.4 Ma or later (Figs. 6 and S2). However, some differences in the pattern were observed among the forms; that is, the forms were roughly classified into those with clear rapid expansion, those with calm expansion, and those with intermediate expansion. Clear rapid expansion was typically shown in clade A of *Gymnogobius isaza* and clade A of *Sarcocheilichthys* spp., whereas the expansion pattern was relatively calm in *Squalidus biwae biwae* (Figs. 5 and S2A). The species in which the null hypothesis of neutrality was not rejected by Fu's *F*_*S*_ exhibited an almost stable population size in BSP (e.g., *Cobitis magnostriata*,* Carassius cuvieri*, and *Silurus lithophilus*; Fig. 5B). The times to the most recent common ancestor (tMRCAs) of most endemic forms and five intraspecific clades (i.e., three clades of *Sarcocheilichthys* spp. and two clades of *G. isaza*) were generally shorter than 0.4 Myr (average ± SD = 0.15 ± 0.09, range 0.03–0.32, *n *=* *13; Table 4, Figs. 6 and S2). On the other hand, the tMRCAs of the species that included two or more distinct intraspecific clades (i.e., *C. buergeri grandoculis*,* C. cuvieri*,* Sarcocheilichthys* spp., and *G. isaza*) were older than 0.4 Myr (average ± SD = 0.88 ± 0.67, range 0.40–1.88, *n *=* *4; Table 4, Figs. 6 and S2).

The BSP analysis of 11 nonendemics also suggested population expansions that began *c*. 0.4 Ma or later (Figs. 6 and S2). The tMRCAs of these nonendemic fishes, except for *Tanakia lanceolata*, were mostly shorter than 0.4 Myr (Table 4, Figs. 6 and S2). The nine nonendemic forms that did not show the population expansion pattern included distinct intraspecific clades (e.g., *Tribolodon hakonensis*,* Silurus asotus*), and the tMRCAs of those species, as with some endemic forms, were longer than 0.4 Myr (Table 4, Figs. 6 and S2).

In several species groups, haplotypes common or very close to those found in Lake Biwa were detected from other riverine populations in western Japan (Fig. S3), suggesting multiple colonizations into Lake Biwa by some intraspecific lineages. For example, both clade A and B haplotypes of *Sarcocheilichthys* spp. were obtained from the Lake Biwa, Kinki, and Chugoku regions, and clade C haplotypes were obtained from the Lake Biwa and Kyushu regions. Common or very close haplotypes of *Carassius buergeri grandoculis* (Lake Biwa endemics) were shared by *C. buergeri* subspp. populations from the Kinki and Kyushu regions of western Japan. Lake Biwa populations of seven nonendemic species (i.e., *Tanakia limbata*,* G. elongatus*,* Pseudogobio esocinus*,* Rhynchocypris oxycephalus jouyi*,* Tribolodon hakonensis*,* Nipponocypris sieboldii*, and *Silurus asotus*) had haplotypes of two or more clades that were shared with the Chugoku, Kinki, and Tokai regions of western Japan.

## Discussion

### Divergence times and origins of endemic fish in Lake Biwa

Based on a Bayesian approach with geological calibrations, the evolutionary rates of each mitochondrial gene were estimated to be ~0.3–1.5% Myr^−1^ lineage^−1^. No discrepancies were found between our rates and those previously reported for each region (e.g., Avise 2000; Kuo et al. 2003; Watanabe and Takahashi 2010). Ho et al. (2005) pointed out an apparent acceleration of the divergence rate over a short time period (<1–2 Myr or ~1% substitution). However, except for a few cases, the divergences of endemic forms from their closest relatives exhibited greater genetic distances (>1% substitution in the uncorrected *P*‐distance) than would occur with such a threshold. Additionally, the estimated rates were within in the range of known molecular clocks of teleost mitochondrial genes (~0.1–2.5% Myr^−1^ lineage^−1^; e.g., Burridge et al. 2008; Watanabe and Takahashi 2010). Therefore, it is reasonable to infer divergence times using these estimated evolutionary rates. Nevertheless, it should be noted that the estimations could be biased toward older divergence times when these long‐term evolutionary rates are used in the analysis of a recent historical demography (Ho et al. 2005) (see the next section).

Bayesian phylogenetic analysis using five mitochondrial genes suggested that seven of the 11 examined endemic species diverged from their closest species long before the origin of the present Lake Biwa environment (~0.4 Ma) (hereafter the “old‐endemic” group), whereas four other species diverged ca. 0.4 Ma (or did not clearly diverge from their closest relatives; hereafter the “new‐endemic” group). Previous studies presumed that endemic fish that had been inferred to be adapted to the present Lake Biwa environment (the rocky substrate, extensive deep pelagic area, etc.) differentiated from their ancestors after the establishment of the particular environments of the lake (e.g., Nakajima 1987; Takahashi 1989). However, many of the endemic species were inferred to have diverged from their closest species long before 0.4 Ma.

It has been inferred that the new‐endemics diverged recently from or maintained gene flow with their closest relatives. No remarkable genetic differentiation was found among *Sarcocheilichthys biwaensis*,* S. variegates microoculus* (endemic to Lake Biwa), and *S. variegates variegates* (distributed widely in rivers in western Japan but not in Lake Biwa) in their mtDNA (Komiya et al. 2011; 2013). The head shape divergence and the rapid speciation in this group in Lake Biwa were inferred to be associated with feeding habits corresponding to substrate types in the lake (i.e., rocky vs. pebbly areas; Komiya et al. 2011; 2013). *Squalidus biwae biwae* (endemic to Lake Biwa) and its closest form *Squalidus biwae tsuchigae* (distributed in the southern basin of Lake Biwa, the rivers flowing into the lake, and around the Seto Inland Sea and Ise Bay regions) exhibit steep but continuous genetic and morphological variations along the environmental gradient around the lake (Kakioka 2013). This could be explained by adaptations in body shape to pelagic life for efficient swimming performance, along with some gene flow. On the other hand, it has been inferred that the origin of *Oncorhynchus masou* subsp. “Lake Biwa trout” diverged a little earlier than those of the above groups (0.52 Ma, 95%HPD: 0.29–0.76 Ma), and a sister relationship between this trout and all other masu trout subspecies (i.e., *Oncorhynchus masou masou*,* Oncorhynchus masou ishikawae*, and *Oncorhynchus masou formosanum*) has been amply supported. The masu trout group basically has an anadromous life history. Our results support that the Lake Biwa trout was differentiated from the ancestral anadromous or landlocked form by its use of the lake instead of the sea after the pelagic zone of the present Lake Biwa began to form (Oohara and Okazaki 1996; Fujioka 2009).

The old‐endemic group was inferred to have originated from lineages that had already diverged from any other existing lineages much earlier than the present Lake Biwa stage (i.e., prior to 0.4 Ma). It has been suggested that those endemic forms exhibit apparent adaptations to the lake environment in terms of their morphologies, behaviors, and life histories (Tomoda 1978; Takahashi 1989). This suggests that those endemic forms originated from lineages different from those of the present riverine closest relatives and that they colonized and adapted to the present lake following the extinction of their direct riverine ancestral populations. This pattern differs from the rapid evolution following adaptation to new environments observed in the cichlids of Lake Victoria (Seehausen 2006; Turner 2007) or the sticklebacks and salmonids (*Coregonus* and *Salvelinus*) of the postglacial lakes of North America and northern Europe (Schluter 1995; Bernatchez et al. 1999; Gíslason et al. 1999).

The older origins of *Gymnogobius isaza*,* Gnathopogon caerulescens*, and *Carassius cuvieri* have been suggested by previous molecular phylogenetic studies based on shorter mtDNA sequences (Harada et al. 2002; Takada et al. 2010; Kakioka et al. 2013, 2015; Tabata and Watanabe 2013). The present study supports these results and demonstrates that *Silurus lithophilus*, which presumably has traits that are adaptive in rocky areas (e.g., anteriorly positioned eyes and yellowish brown body color), also has an older origin, and its sister group is a cryptic lineage that has been included in *Silurus asotus* (see Table 3, Fig. 3). *Cottus reinii* (lacustrine type) (a cottoid, landlocked and presumably adapted to the bottom of the deep area) and *Opsariichthys. uncirostris* (a cyprinid, regarded as a “relict species” because of the absence of extant relative species in Japan) were also inferred to have diverged from their closest forms before the establishment of the present Lake Biwa. *Silurus biwaensis* was confirmed to have distant relationships with the two other silurids and is considered a relict. Although the endemic goby *Rhinogobius* sp. BW was excluded from our analysis because of suspected mtDNA introgression, Yamasaki et al. (2015) showed that the species is also included in the old‐endemic group (divergence ca. 0.9–1.1 Ma from its closest relative) using multiple nuclear genes.

The divergence times of the old‐endemic lineages from their closest lineages ranged from about 1.3 Myr (*C. reinii* lacustrine type–a lineage of *C. reinii* amphidromous type) to 3.0 Myr (*O. uncirostris*–*O. bidens*), which are mainly in the early Pleistocene (Fig. 4). One possible explanation is the Pleistocene climate changes accompanying global cooling and drying in the glacial periods (Provan and Bennett 2008), which could affect the divergence and speciation of the endemic species found in the present Lake Biwa. Although western Japan has never been covered with ice sheets, such climatic changes caused remarkable changes in the freshwater environment and may have led to the extinction of species (Lindberg 1972; Clark et al. 1999; Provan and Bennett 2008). In addition, such environmental changes, together with drastic crustal movements (e.g., uplifting of mountains), would have promoted the population isolation and differentiation as well as the adaptation to novel environments of freshwater fish.

The possible important roles played by Paleo‐Lake Biwa in the divergence and adaptation of endemic species should be taken into consideration. Paleo‐Lake Biwa is the historical series of lake or marsh environments that existed from *c*. 4 Ma (the Pliocene) to 0.4 Ma, prior to the present Lake Biwa (Yokoyama 1984; Kawabe 1989, 1994). After the Pliocene, the topographical and limnological features of Paleo‐Lake Biwa (and Lake Tokai, located east of Paleo‐Lake Biwa) successively changed: the paleo‐lake experienced (1) shallow‐and‐large stages (“Lake Ohyamada,” >3.2 Ma; “Lake Ayama,” 3.0–2.7 Ma); (2) a deep‐and‐large stage (“Lake Koka,” 2.7–2.5 Ma), similar to the northern basin of the present Lake Biwa; (3) a second shallow‐and‐large stage (“Lake Gamo,” 2.5–1.8 Ma); (4) a swamp‐like stage (1.8–1.0 Ma); and (5) a final shallow‐and‐large stage (“Lake Katata,” 1.0–0.5 Ma) (Kawabe 1989, 1994). Past lakes and river systems have contributed to the present species richness and distribution in some cases. The foundation of the diversity of present cichlids, generated in Paleo‐Lake Makgadikgadi in Africa (Joyce et al. 2005), and the distribution of *Barbus* in the Balkan Peninsula (Marková et al. 2010) have been cited as examples. Although the confidence intervals of estimated divergence times are wide, the divergences of the old‐endemic lineages were likely concentrated between 2.0 and 3.0 Ma, that is, during the “Lake Koka” stage. It might be that Lake Koka, which existed as a deep‐and‐large lake, induced the divergence with adaptation of the ancestral populations of the extant species to the limnetic environment (Harada et al. 2002). During the subsequent desiccated periods, the alleles that had adapted to the paleo‐lake might have been retained as standing genetic variations in the populations that survived in rivers or marshes, as was the case with the cichlids in Lake Malawi and Lake Victoria (Elmer et al. 2009; Loh et al. 2013).

The mtDNA loci consist of a single linkage group; hence, they might cause a biased estimation of phylogenetic relationships and divergence times due to discrepancies between mtDNA and nuclear DNA trees and/or between gene trees and species trees (Avise 2000; Edwards and Beerli 2000; Walstrom et al. 2012). Our estimations, therefore, must be tested with a multilocus nuclear DNA dataset. Furthermore, studies on genetic backgrounds and the origins of adaptive genetic variations to lake environments using evolutionary genomic approaches are necessary (Gilad et al. 2009; Schoville et al. 2012; Bernardi 2013).

### Historical demography and establishment of the ichthyofauna in the present lake

The demographic history inferred based on mtDNA diversity suggests that most of the present fish species in Lake Biwa experienced some historical bottleneck or founder effect after the present Lake Biwa environment began to develop. That is, the mismatch distribution and BSP analyses revealed expansion patterns of the populations (more precisely, of the mitochondrial effective population sizes) after 0.3 Ma (predominantly after 0.15 Ma) in more than half the analyzed fish species in Lake Biwa, including the old‐ and new‐endemics. In addition, the tMRCAs of almost all of the examined fish populations were estimated to be <0.4 Myr. These figures might be biased toward earlier dates because the molecular evolutionary rates used were calibrated by older events and hence they might be too small to apply to recent population histories (Ho et al. 2005). Even in such cases, our results regarding the expansion in recent geological periods are still supported. These demographic patterns suggest drastic changes and the establishment of the ichthyofauna during the present Lake Biwa stage.

The new‐endemic species in Lake Biwa are inferred to have expanded their populations following divergences from their closest species (or subspecies). The old‐endemics, on the other hand, had already diverged from their closest relatives long before the present Lake Biwa stage (prior to 0.4 Ma) and had colonized present Lake Biwa. The latter species might have acquired their presently observed phenotypic characteristics by adapting to the lacustrine environment after the colonization of their ancestral populations. That is, there would have been a time lag between the phylogenetic divergences of the endemic lineages and the acquisition (or reacquisition) of their present adaptive traits. Such a time lag might be explained by rapid phenotypic acquisition (or reacquisition) through strong selective pressure when the fish entered the novel limnetic environments.

Alternatively, the reduction and rapid expansion of the mtDNA effective population size might not reflect the dynamics of actual population size but instead might have resulted from a selective sweep followed by the recovery of genetic diversity in mtDNA. The mitochondrion is an important organelle that bears genes related to aerobic respiration and energy production that are involved in exercise and metabolism. Therefore, the mtDNA genome (and nuclear genes related to mitochondrial functions) can be a target of natural selection (e.g., Doiron et al. 2002; Hurst and Jiggins 2005; Ballard and Melvin 2010; Silva et al. 2014); i.e., natural selection might have favored improvements in exercise and metabolic capacity, when fish colonized the large pelagic area or the hypoxic, low‐temperature bottom area in the present Lake Biwa. Selection for genetic variation of a single gene in a mitochondrial genome could cause a selective sweep of an entire mitochondrial genome due to the lack of recombination; this could manifest as a pattern similar to a population bottleneck. However, selective sweep alone would not be able to explain the entire expansion pattern because the pattern was observed not only in endemic species but also in other species around the lake. The historical population demography of Lake Biwa fish should be reexamined using multiple, highly variable nuclear DNA loci to differentiate selective sweep caused by selection on a single locus.

### The roles of Lake Biwa in the formation of ichthyofauna in western Japan

Lake Biwa is presumed to have functioned not only as the center of adaptive evolution and speciation but also as an important refugium (or “reservoir”) for freshwater fish in western Japan since the middle Pleistocene because of its huge capacity and historical stability. In some endemic and non‐endemic species, common or very close haplotypes were shared between Lake Biwa and rivers/lakes in other regions of western Japan. This suggests some historical gene flow between populations in these regions as well as secondary contact following multiple colonizations in Lake Biwa (Fig. S3). This situation differs from the cases in other typical ancient lakes, such as the African Great Lakes and Lake Baikal, with much higher rates of endemic species (>80% vs. <25% in Lake Biwa). The divergent mtDNA lineages within the endemic population of *Gymnogobius isaza* also support the secondary contact in Lake Biwa between past divergent populations (Tabata and Watanabe 2013; present study). Global climate changes (e.g. Clark et al. 1999; Provan and Bennett 2008) and drastic changes in inland water environments in the Pleistocene (Bernatchez and Wilson 1998; Yokoyama et al. 2000) would have resulted in the reduction or extinction of many freshwater fish populations in western Japan (Watanabe and Takahashi 2010). Such extinctions outside Lake Biwa would be evidenced by some relict species in the lake, such as *Opsariichthys uncirostris* and *Ischikauia steenackeri*, whose distributions have been expanded through artificial introduction (Sato et al. 2010; Watanabe 2012). The significantly lower genetic diversity in riverine populations compared with the Lake Biwa population of *Pseudobagrus nudiceps* may also support the severe riverine conditions in the past (Watanabe and Nishida 2003). The role of Lake Biwa as a reservoir of species and genetic diversity and, probably, as a source of dispersal, would have contributed to the formation and maintenance of ichthyofauna in western Japan.

The faunal history of Lake Biwa involving the absorption and accumulation of divergent lineages within species may have also contributed to the evolvability of Lake Biwa populations by increasing and preserving genetic diversity. Unlike those in the high‐latitude lakes of Europe and North America that were severely affected by glaciations (Avise 2000; Hewitt 2000, 2001), the ichthyofauna in the temperate zone of western Japan may have continuously developed since the Pliocene (e.g., Hewitt 2004; Watanabe and Takahashi 2010). The lake would have functioned as a biogeographic reservoir not only in the present Lake Biwa but also in the Paleo‐Lake Biwa stages. Standing genetic variations accumulated in the long history of Lake Biwa may have promoted rapid evolutionary responses in changing lake environments (Renaut et al. 2011; Kakioka et al. 2013, 2015). The new insights on the origin of the endemic ichthyofauna of Lake Biwa obtained through this study will be an important starting point for progressive studies of adaptive evolution in this ancient lake.

## Conflict of Interest

None declared.

## Supporting information


**Figure S1.** Bayesian phylogenetic trees of each fish group, including outgroup species, based on mtDNA 16S, CO1, ND5 cyt *b*, and CR gene sequences.

**Figure S2. (A)** Mismatch distributions and statistical parsimony networks of mtDNA cytochrome *b* haplotypes of each species. **(B)** The results of Bayesian skyline plots for fishes in Lake Biwa.

**Figure S3.** Statistical parsimony networks of mtDNA cytochrome *b* haplotypes of selected fishes including Lake Biwa and other local populations.

**Figure S4.** Distribution maps of the of Japanese freshwater fishes used in this study. The species/subspecies with an asterisk are the closest Lake Biwa endemics.

**Table S1.** List of data used for the phylogenetic and population genetic analyses and sampling sites.
**Table S2.** Localities and sample sizes of fishes in Lake Biwa and the rivers around the lake used for the population genetic analyses.
**Table S3.** The data used for the phylogeographic analyses, excluding Lake Biwa populations.
**Table S4.** List of PCR and sequencing primers.
**Table S5.** List of substitution models selected by the Bayesian information criterion implemented in jModeltest 2.1.1 and the estimated mean mutation rate of each locus.
**Table S6.** Results of mismatch distribution analyses and neutrality tests.
