# Supplementary material for: Phylogeny and historical demography of endemic fishes in Lake Biwa: the ancient lake as a promoter of evolution and diversification of freshwater fishes in western Japan
Source: Ecol Evol. 2016 Mar 16;6(8):2601–23. doi: 10.1002/ece3.2070 (PMC4798153; doi:10.1002/ece3.2070)
Supplement: Supplementary file 1 — Figure S1. Bayesian phylogenetic trees of each fish group, including outgroup species, based on mtDNA 16S, CO1, ND5 cyt b, and CR gene sequences. [file ECE3-6-2601-s001.pdf]

Salmonidae

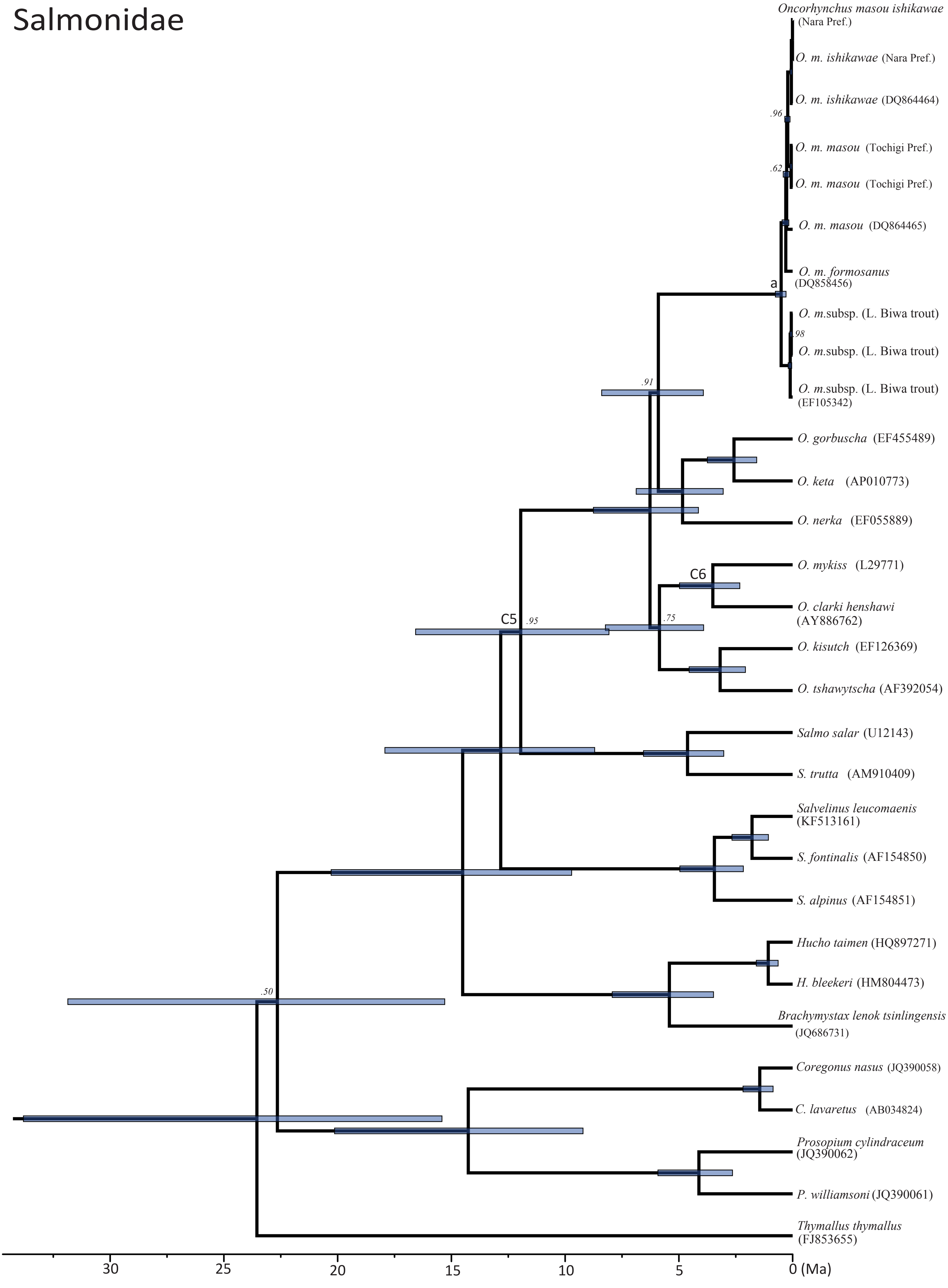

Oncorhynchus masou ishikawae (Nara Pref.)
O. m. ishikawae (Nara Pref.)
O. m. ishikawae (DQ864464)
.96
O. m. masou (Tochigi Pref.)
.62
O. m. masou (Tochigi Pref.)
O. m. masou (DQ864465)
O. m. formosanus (DQ858456)
a
O. m.subsp. (L. Biwa trout)
.98
O. m.subsp. (L. Biwa trout)
O. m.subsp. (L. Biwa trout) (EF105342)
.91
O. gorbuscha (EF455489)
O. keta (AP010773)
O. nerka (EF055889)
C6
O. mykiss (L29771)
O. clarki henshawi (AY886762)
C5
.95
.75
O. kisutch (EF126369)
O. tshawytscha (AF392054)
Salmo salar (U12143)
S. trutta (AM910409)
Salvelinus leucomaenis (KF513161)
S. fontinalis (AF154850)
S. alpinus (AF154851)
Hucho taimen (HQ897271)
H. bleekeri (HM804473)
.50
Brachymystax lenok tsinlingensis (JQ686731)
Coregonus nasus (JQ390058)
C. lavaretus (AB034824)
Prosopium cylindraceum (JQ390062)
P. williamsoni (JQ390061)
Thymallus thymallus (FJ853655)
30
25
20
15
10
5
0 (Ma)

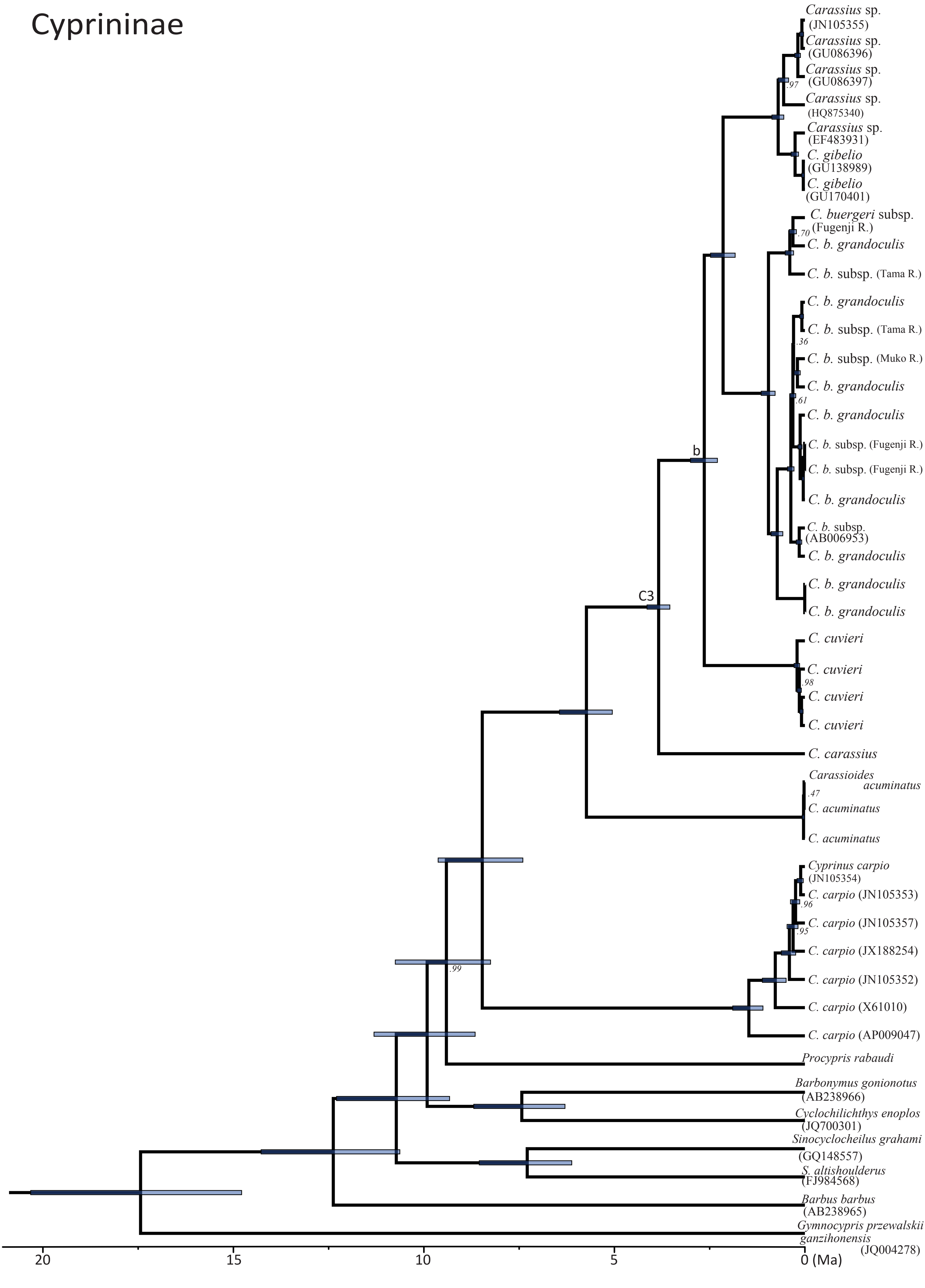

Cyprininae
Carassius sp. (JN105355)
Carassius sp. (GU086396)
Carassius sp. (GU086397)
.97
Carassius sp. (HQ875340)
Carassius sp. (EF483931)
C. gibelio (GU138989)
C. gibelio (GU170401)
C. buergeri subsp. (Fugenji R.)
.70
C. b. grandoculis
C. b. subsp. (Tama R.)
C. b. grandoculis
C. b. subsp. (Tama R.)
.36
C. b. subsp. (Muko R.)
C. b. grandoculis
.61
C. b. grandoculis
C. b. subsp. (Fugenji R.)
C. b. subsp. (Fugenji R.)
C. b. grandoculis
C. b. subsp. (AB006953)
C. b. grandoculis
C. b. grandoculis
C. b. grandoculis
b
C3
C. cuvieri
C. cuvieri
.98
C. cuvieri
C. cuvieri
C. carassius
Carassioides acuminatus
.47
C. acuminatus
C. acuminatus
Cyprinus carpio (JN105354)
C. carpio (JN105353)
.96
C. carpio (JN105357)
.95
C. carpio (JX188254)
C. carpio (JN105352)
C. carpio (X61010)
C. carpio (AP009047)
.99
Procypris rabaudi
Barbonymus gonionotus (AB238966)
Cyclochilichthys enoplos (JQ700301)
Sinocyclocheilus grahami (GQ148557)
S. altishoulderus (FJ984568)
Barbus barbus (AB238965)
Gymnocypris przewalskii ganzihonensis (JQ004278)
20
15
10
5
0 (Ma)

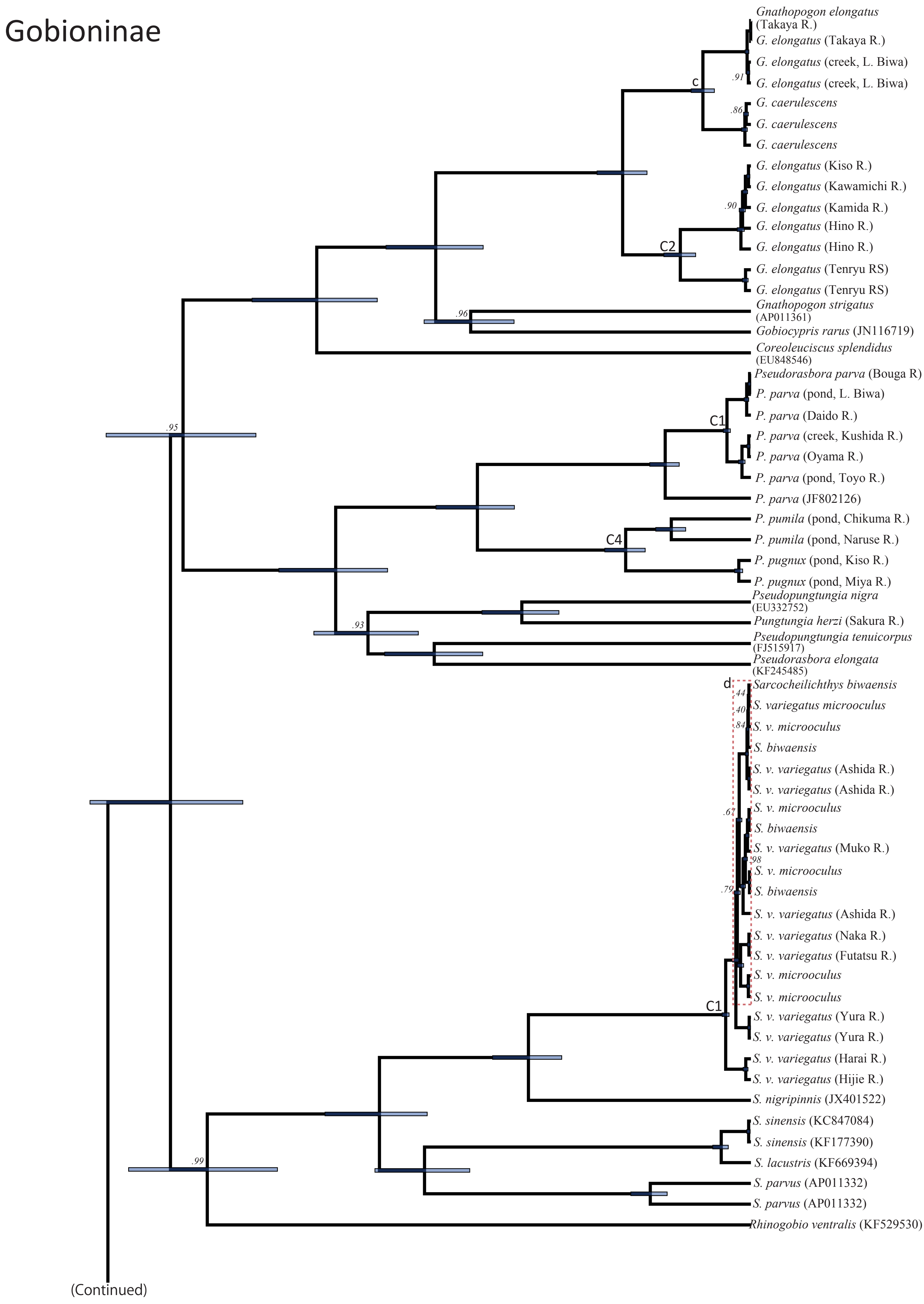
Gobioninae
Gnathopogon elongatus (Takaya R.)
G. elongatus (Takaya R.)
G. elongatus (creek, L. Biwa)
.91
G. elongatus (creek, L. Biwa)
c
G. caerulescens
.86
G. caerulescens
G. caerulescens
G. elongatus (Kiso R.)
G. elongatus (Kawamichi R.)
.90
G. elongatus (Kamida R.)
G. elongatus (Hino R.)
C2
G. elongatus (Hino R.)
G. elongatus (Tenryu RS)
G. elongatus (Tenryu RS)
Gnathopogon strigatus (AP011361)
.96
Gobiocypris rarus (JN116719)
Coreoleuciscus splendidus (EU848546)
.95
Pseudorasbora parva (Bouga R)
P. parva (pond, L. Biwa)
P. parva (Daido R.)
C1
P. parva (creek, Kushida R.)
P. parva (Oyama R.)
P. parva (pond, Toyo R.)
P. parva (JF802126)
P. pumila (pond, Chikuma R.)
C4
P. pumila (pond, Naruse R.)
P. pugnux (pond, Kiso R.)
P. pugnux (pond, Miya R.)
Pseudopungtungia nigra (EU332752)
.93
Pungtungia herzi (Sakura R.)
Pseudopungtungia tenuicorpus (FJ515917)
Pseudorasbora elongata (KF245485)
d
Sarcocheilichthys biwaensis
.44
S. variegatus microoculus
.40
.84
S. v. microoculus
S. biwaensis
S. v. variegatus (Ashida R.)
S. v. variegatus (Ashida R.)
.67
S. v. microoculus
S. biwaensis
S. v. variegatus (Muko R.)
.98
S. v. microoculus
.79
S. biwaensis
S. v. variegatus (Ashida R.)
S. v. variegatus (Naka R.)
S. v. variegatus (Futatsu R.)
S. v. microoculus
S. v. microoculus
C1
S. v. variegatus (Yura R.)
S. v. variegatus (Yura R.)
S. v. variegatus (Harai R.)
S. v. variegatus (Hijie R.)
S. nigripinnis (JX401522)
S. sinensis (KC847084)
S. sinensis (KF177390)
S. lacustris (KF669394)
S. parvus (AP011332)
.99
S. parvus (AP011332)
Rhinogobio ventralis (KF529530)
(Continued)

(Continued)

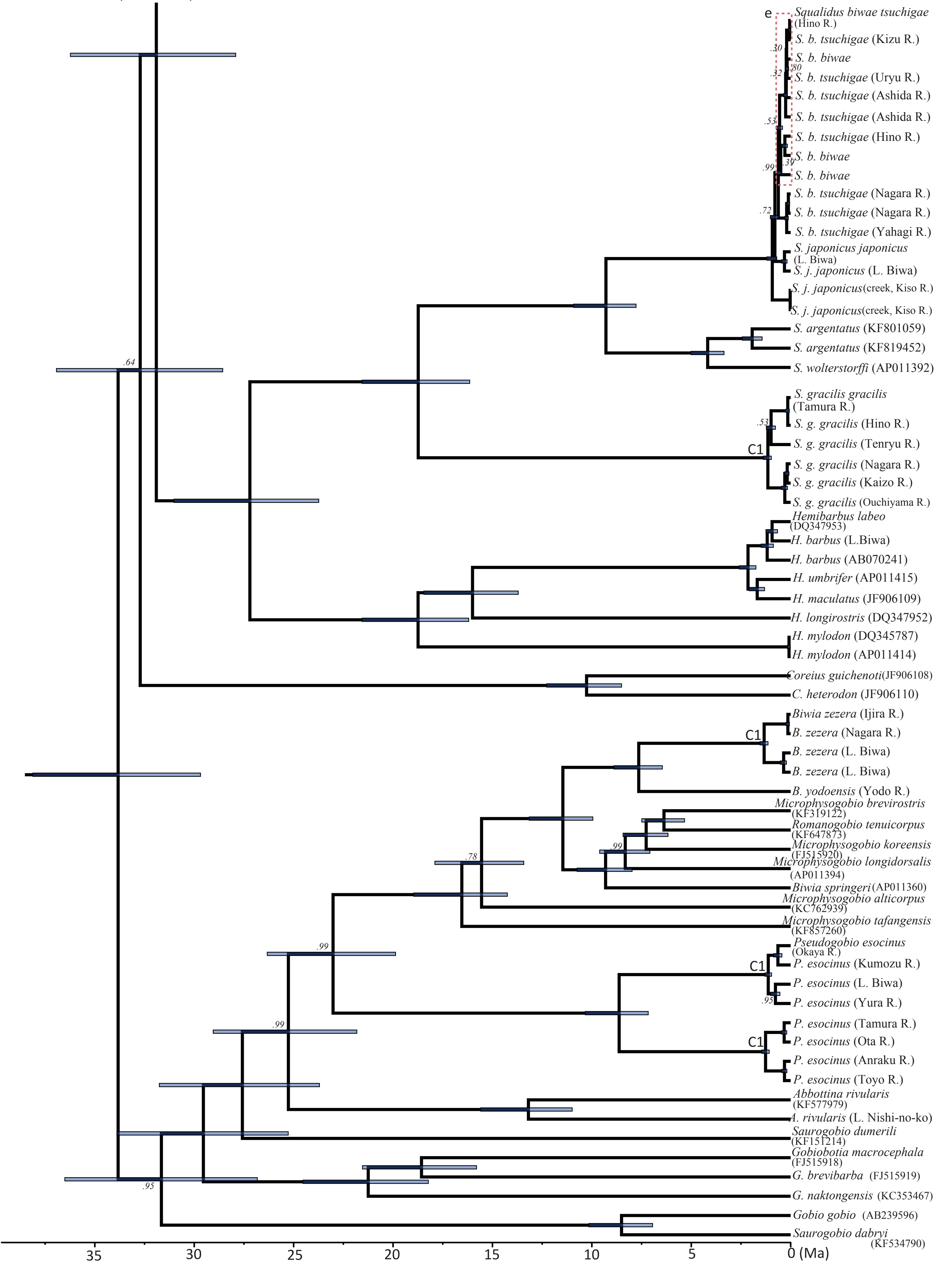
e
Squalidus biwae tsuchigae (Hino R.)
S. b. tsuchigae (Kizu R.)
.30
S. b. biwae
.80
.32
S. b. tsuchigae (Uryu R.)
S. b. tsuchigae (Ashida R.)
S. b. tsuchigae (Ashida R.)
.53
S. b. tsuchigae (Hino R.)
S. b. biwae
.39
.99
S. b. biwae
S. b. tsuchigae (Nagara R.)
S. b. tsuchigae (Nagara R.)
.72
S. b. tsuchigae (Yahagi R.)
S. japonicus japonicus (L. Biwa)
S. j. japonicus (L. Biwa)
S. j. japonicus (creek, Kiso R.)
S. j. japonicus (creek, Kiso R.)
S. argentatus (KF801059)
S. argentatus (KF819452)
S. wolterstorffi (AP011392)
.64
S. gracilis gracilis (Tamura R.)
.53
S. g. gracilis (Hino R.)
S. g. gracilis (Tenryu R.)
C1
S. g. gracilis (Nagara R.)
S. g. gracilis (Kaizo R.)
S. g. gracilis (Ouchiyama R.)
Hemibarbus labeo (DQ347953)
H. barbus (L.Biwa)
H. barbus (AB070241)
H. umbrifer (AP011415)
H. maculatus (JF906109)
H. longirostris (DQ347952)
H. mylodon (DQ345787)
H. mylodon (AP011414)
Coreius guichenoti (JF906108)
C. heterodon (JF906110)
Biwia zezera (Ijira R.)
B. zezera (Nagara R.)
C1
B. zezera (L. Biwa)
B. zezera (L. Biwa)
B. yodoensis (Yodo R.)
Microphysogobio brevirostris (KF319122)
Romanogobio tenuicorpus (KF647873)
Microphysogobio koreensis (FJ515920)
.99
Microphysogobio longidorsalis (AP011394)
.78
Biwia springeri (AP011360)
Microphysogobio alticorpus (KC762939)
Microphysogobio tafangensis (KF857260)
.99
Pseudogobio esocinus (Okaya R.)
C1
P. esocinus (Kumozu R.)
P. esocinus (L. Biwa)
.95
P. esocinus (Yura R.)
.99
P. esocinus (Tamura R.)
P. esocinus (Ota R.)
C1
P. esocinus (Anraku R.)
P. esocinus (Toyo R.)
Abbottina rivularis (KF577979)
A. rivularis (L. Nishi-no-ko)
Saurogobio dumerili (KF151214)
Gobiobotia macrocephala (FJ515918)
G. brevibarba (FJ515919)
.95
G. naktongensis (KC353467)
Gobio gobio (AB239596)
Saurogobio dabryi (KF534790)
35
30
25
20
15
10
5
0 (Ma)

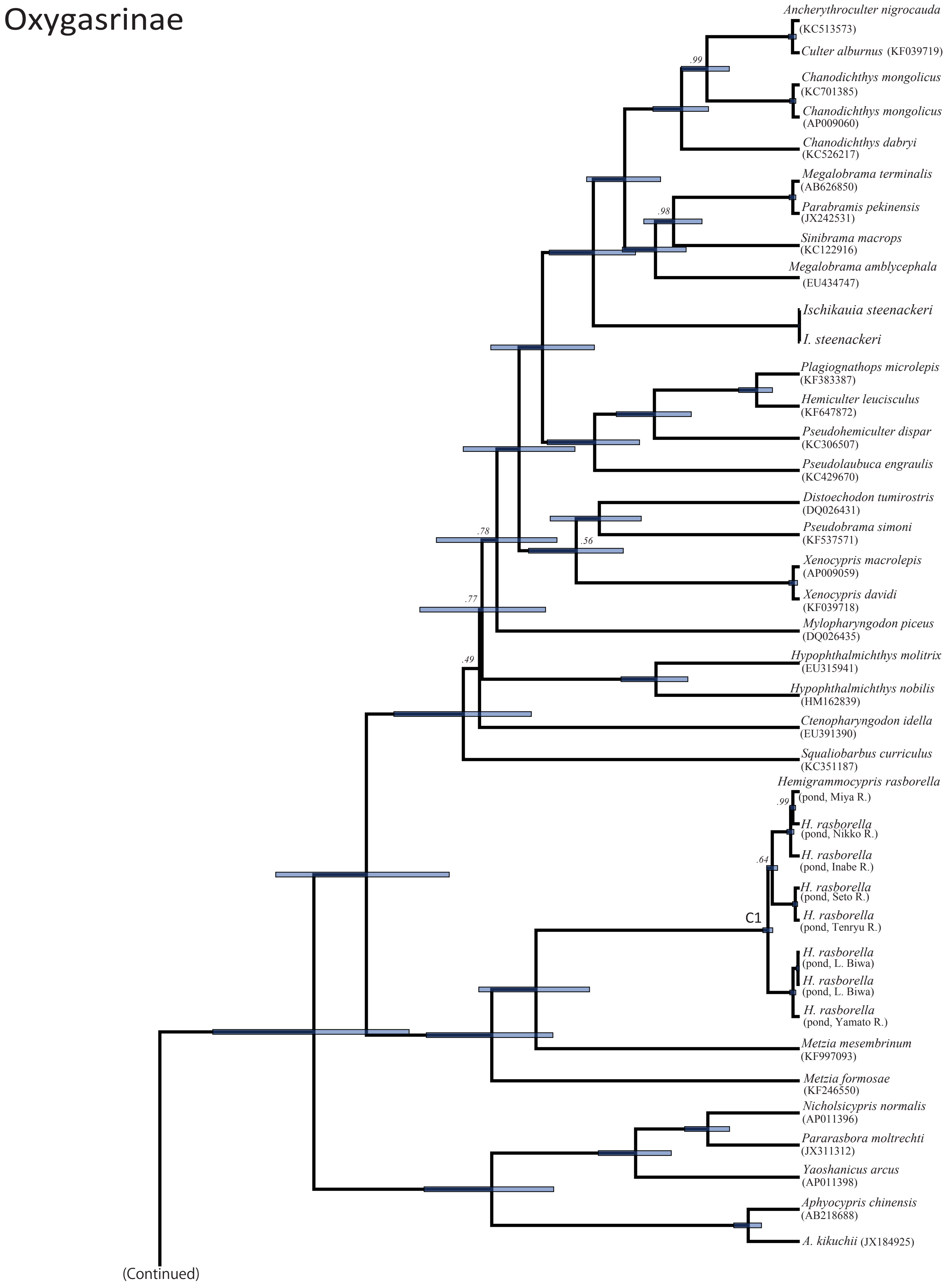
Oxygasrinae
Ancherythroculter nigrocauda (KC513573)
Culter alburnus (KF039719)
.99
Chanodichthys mongolicus (KC701385)
Chanodichthys mongolicus (AP009060)
Chanodichthys dabryi (KC526217)
Megalobrama terminalis (AB626850)
.98
Parabramis pekinensis (JX242531)
Sinibrama macrops (KC122916)
Megalobrama amblycephala (EU434747)
Ischikauia steenackeri
I. steenackeri
Plagiognathops microlepis (KF383387)
Hemiculter leucisculus (KF647872)
Pseudohemiculter dispar (KC306507)
Pseudolaubuca engraulis (KC429670)
Distoechodon tumirostris (DQ026431)
Pseudobrama simoni (KF537571)
.78
.56
Xenocypris macrolepis (AP009059)
Xenocypris davidi (KF039718)
.77
Mylopharyngodon piceus (DQ026435)
.49
Hypophthalmichthys molitrix (EU315941)
Hypophthalmichthys nobilis (HM162839)
Ctenopharyngodon idella (EU391390)
Squaliobarbus curriculus (KC351187)
Hemigrammocypris rasborella (pond, Miya R.)
.99
H. rasborella (pond, Nikko R.)
H. rasborella (pond, Inabe R.)
.64
H. rasborella (pond, Seto R.)
C1
H. rasborella (pond, Tenryu R.)
H. rasborella (pond, L. Biwa)
H. rasborella (pond, L. Biwa)
H. rasborella (pond, Yamato R.)
Metzia mesembrinum (KF997093)
Metzia formosae (KF246550)
Nicholsicypris normalis (AP011396)
Pararasbora moltrechti (JX311312)
Yaoshanicus arcus (AP011398)
Aphyocypris chinensis (AB218688)
A. kikuchii (JX184925)
(Continued)

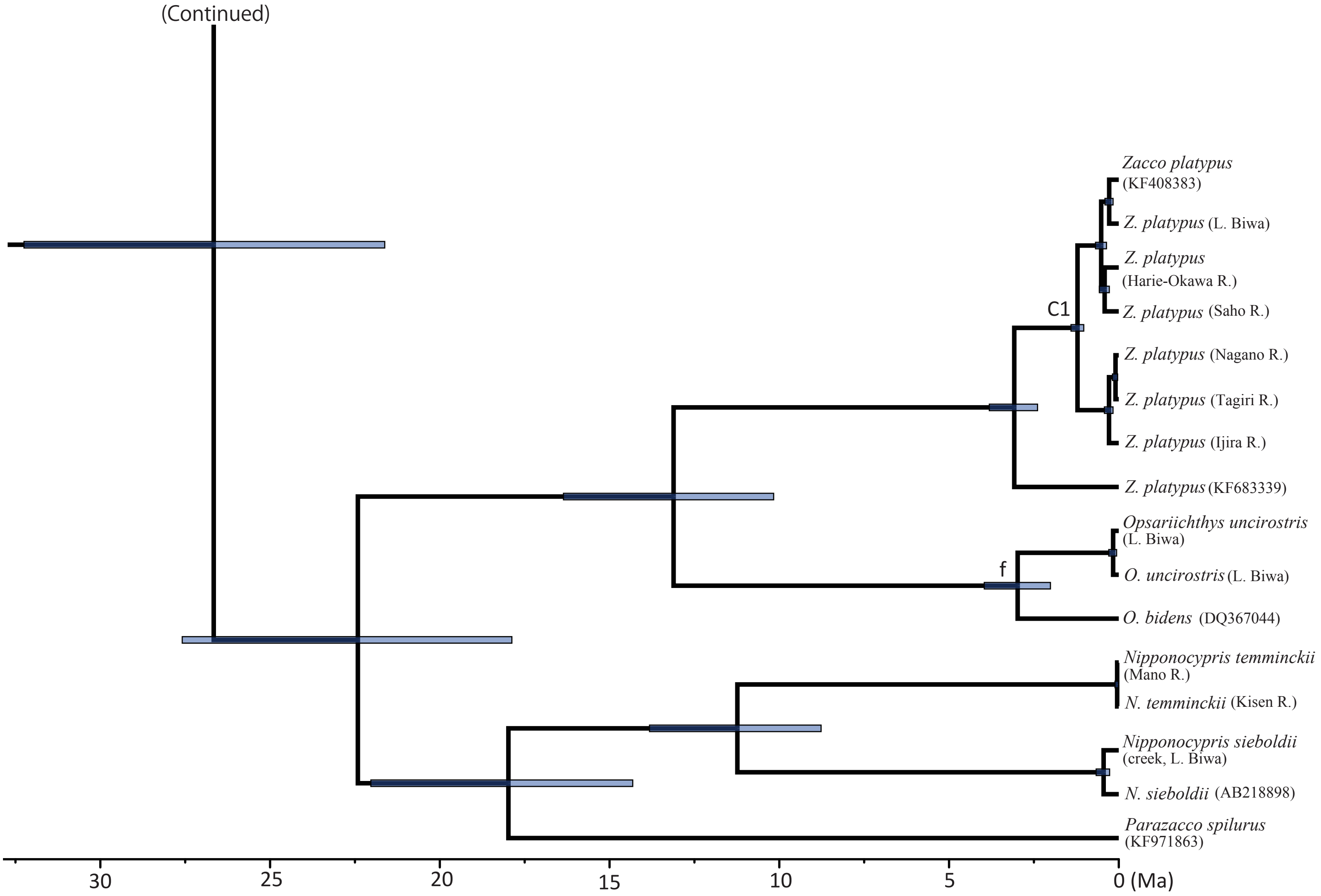

(Continued)
Zacco platypus (KF408383)
Z. platypus (L. Biwa)
Z. platypus (Harie-Okawa R.)
Z. platypus (Saho R.)
C1
Z. platypus (Nagano R.)
Z. platypus (Tagiri R.)
Z. platypus (Ijira R.)
Z. platypus (KF683339)
Opsariichthys uncirostris (L. Biwa)
f
O. uncirostris (L. Biwa)
O. bidens (DQ367044)
Nipponocypris temminckii (Mano R.)
N. temminckii (Kisen R.)
Nipponocypris sieboldii (creek, L. Biwa)
N. sieboldii (AB218898)
Parazacco spilurus (KF971863)
30
25
20
15
10
5
0 (Ma)

Siluridae

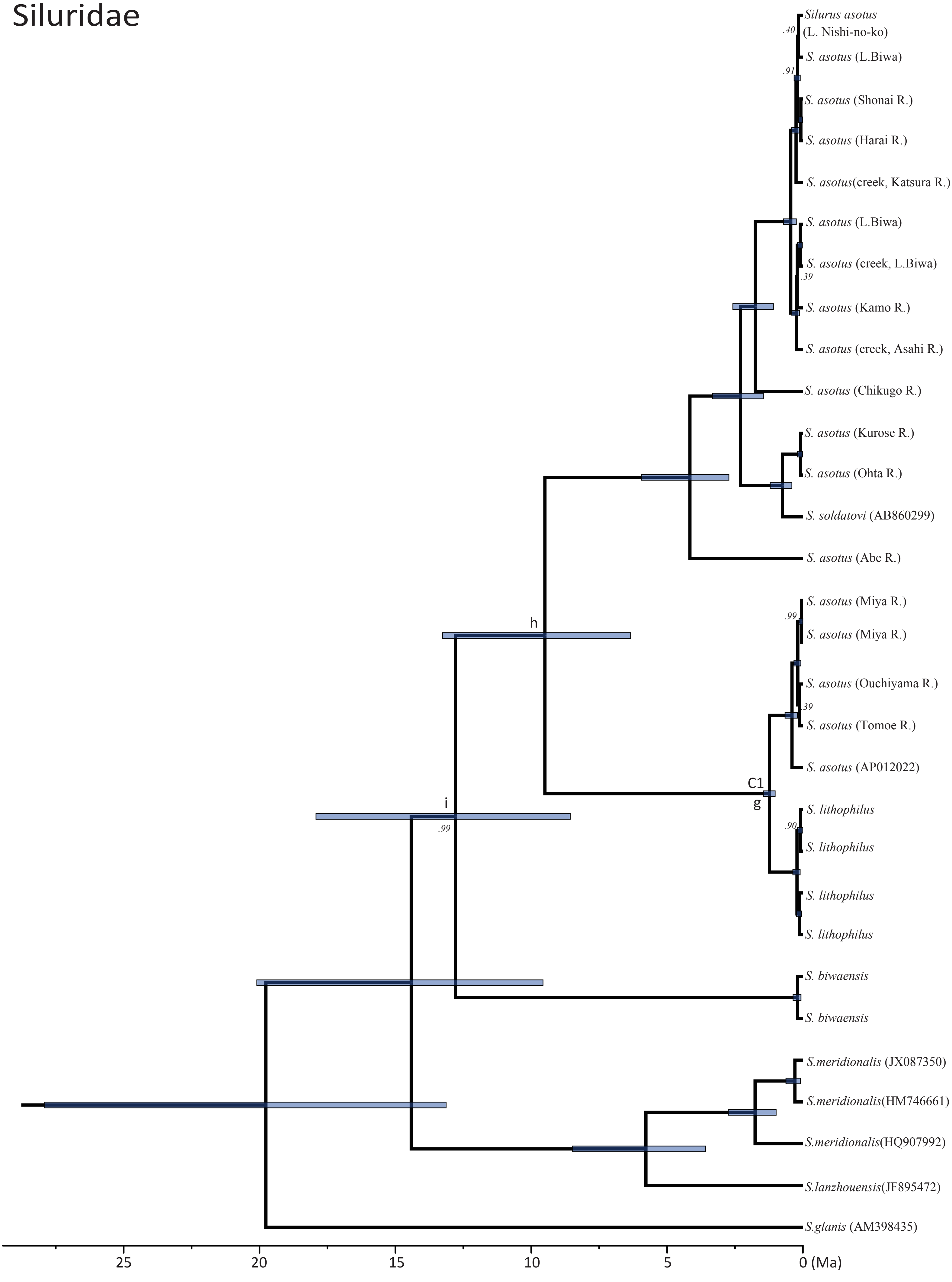

Silurus asotus (L. Nishi-no-ko)
.40
S. asotus (L.Biwa)
.91
S. asotus (Shonai R.)
S. asotus (Harai R.)
S. asotus(creek, Katsura R.)
S. asotus (L.Biwa)
S. asotus (creek, L.Biwa)
.39
S. asotus (Kamo R.)
S. asotus (creek, Asahi R.)
S. asotus (Chikugo R.)
S. asotus (Kurose R.)
S. asotus (Ohta R.)
S. soldatovi (AB860299)
S. asotus (Abe R.)
S. asotus (Miya R.)
.99
S. asotus (Miya R.)
h
S. asotus (Ouchiyama R.)
.39
S. asotus (Tomoe R.)
S. asotus (AP012022)
C1
g
i
.99
S. lithophilus
.90
S. lithophilus
S. lithophilus
S. lithophilus
S. biwaensis
S. biwaensis
S.meridionalis (JX087350)
S.meridionalis(HM746661)
S.meridionalis(HQ907992)
S.lanzhouensis(JF895472)
S.glanis (AM398435)
25
20
15
10
5
0 (Ma)

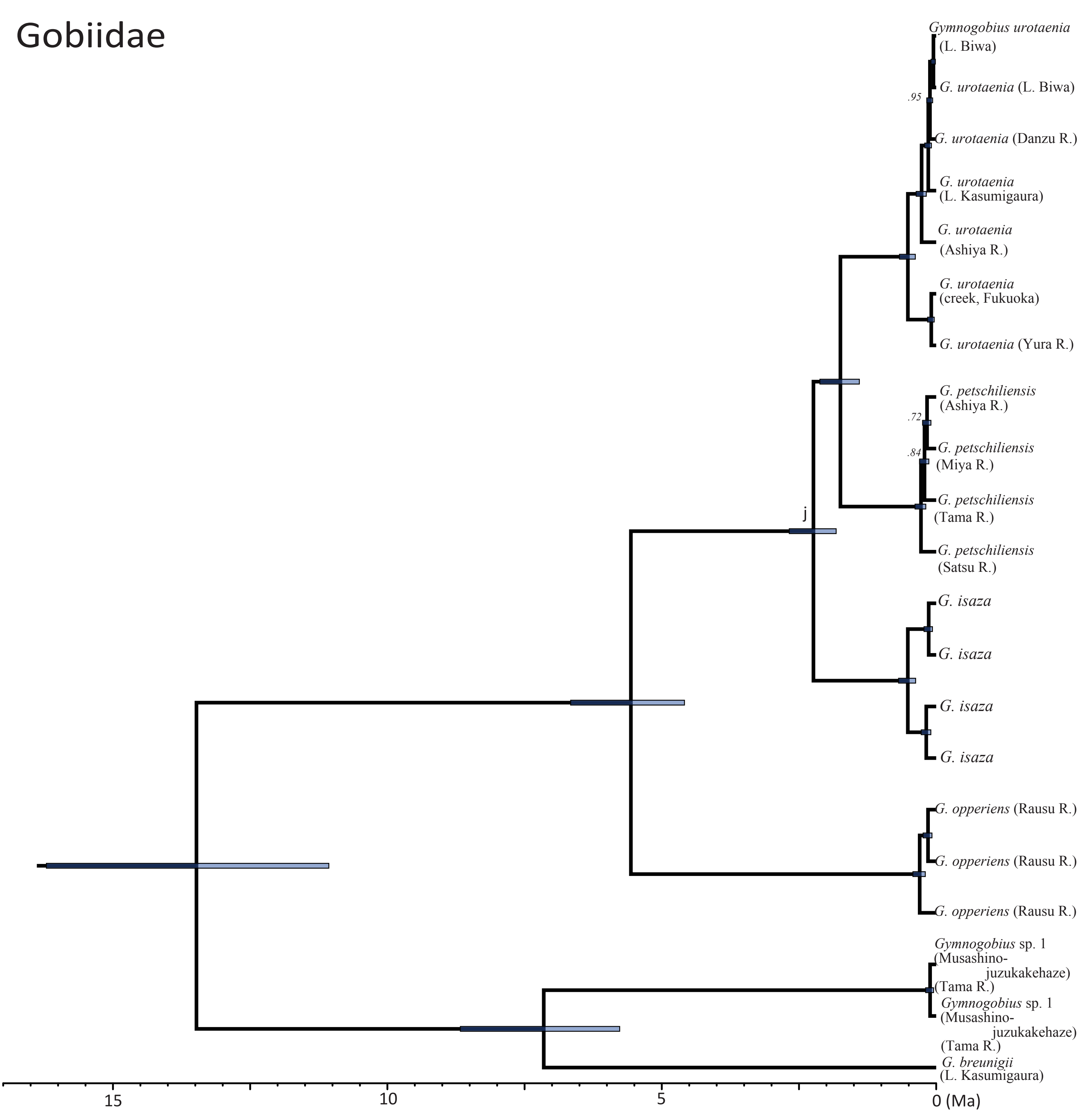

Gobiidae
Gymnogobius urotaenia (L. Biwa)
G. urotaenia (L. Biwa)
.95
G. urotaenia (Danzu R.)
G. urotaenia (L. Kasumigaura)
G. urotaenia (Ashiya R.)
G. urotaenia (creek, Fukuoka)
G. urotaenia (Yura R.)
G. petschiliensis (Ashiya R.)
.72
G. petschiliensis (Miya R.)
.84
G. petschiliensis (Tama R.)
G. petschiliensis (Satsu R.)
j
G. isaza
G. isaza
G. isaza
G. isaza
G. opperiens (Rausu R.)
G. opperiens (Rausu R.)
G. opperiens (Rausu R.)
Gymnogobius sp. 1 (Musashino-juzukakehaze) (Tama R.)
Gymnogobius sp. 1 (Musashino-juzukakehaze) (Tama R.)
G. breunigii (L. Kasumigaura)
15
10
5
0 (Ma)

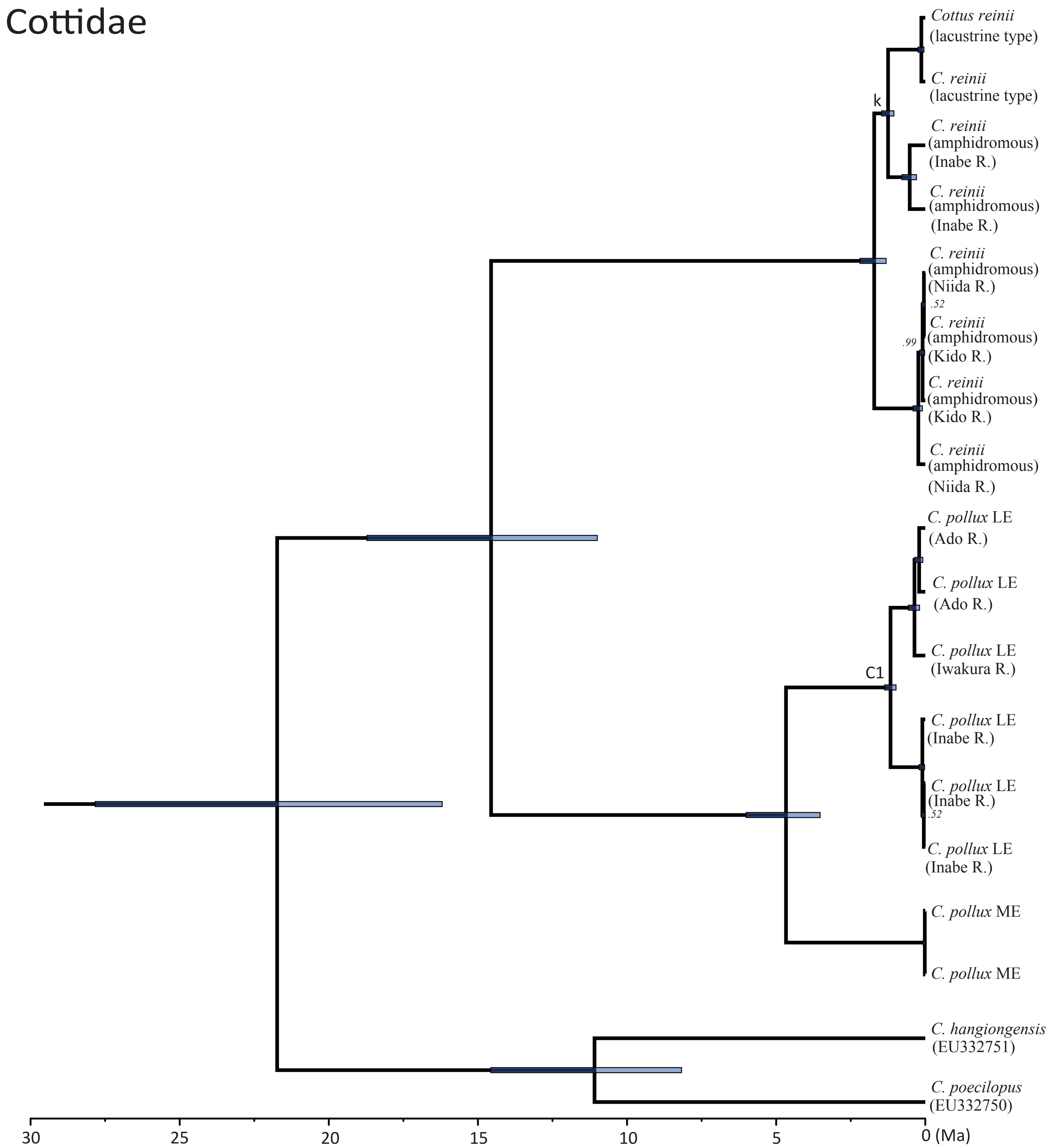
Cottidae
Cottus reinii
(lacustrine type)
C. reinii
(lacustrine type)
k
C. reinii
(amphidromous)
(Inabe R.)
C. reinii
(amphidromous)
(Inabe R.)
C. reinii
(amphidromous)
(Niida R.)
.52
C. reinii
(amphidromous)
.99
(Kido R.)
C. reinii
(amphidromous)
(Kido R.)
C. reinii
(amphidromous)
(Niida R.)
C. pollux LE
(Ado R.)
C. pollux LE
(Ado R.)
C. pollux LE
(Iwakura R.)
C1
C. pollux LE
(Inabe R.)
C. pollux LE
(Inabe R.)
.52
C. pollux LE
(Inabe R.)
C. pollux ME
C. pollux ME
C. hangiongensis
(EU332751)
C. poecilopus
(EU332750)
30
25
20
15
10
5
0 (Ma)

Fig. S1
