# Supplementary material for: Phylogeny and historical demography of endemic fishes in Lake Biwa: the ancient lake as a promoter of evolution and diversification of freshwater fishes in western Japan
Source: Ecol Evol. 2016 Mar 16;6(8):2601–23. doi: 10.1002/ece3.2070 (PMC4798153; doi:10.1002/ece3.2070)
Supplement: Supplementary file 2 — Figure S2. (A) Mismatch distributions and statistical parsimony networks of mtDNA cytochrome b haplotypes of each species. (B) The results of Bayesian skyline plots for fishes in Lake Biwa. [file ECE3-6-2601-s002.pdf]

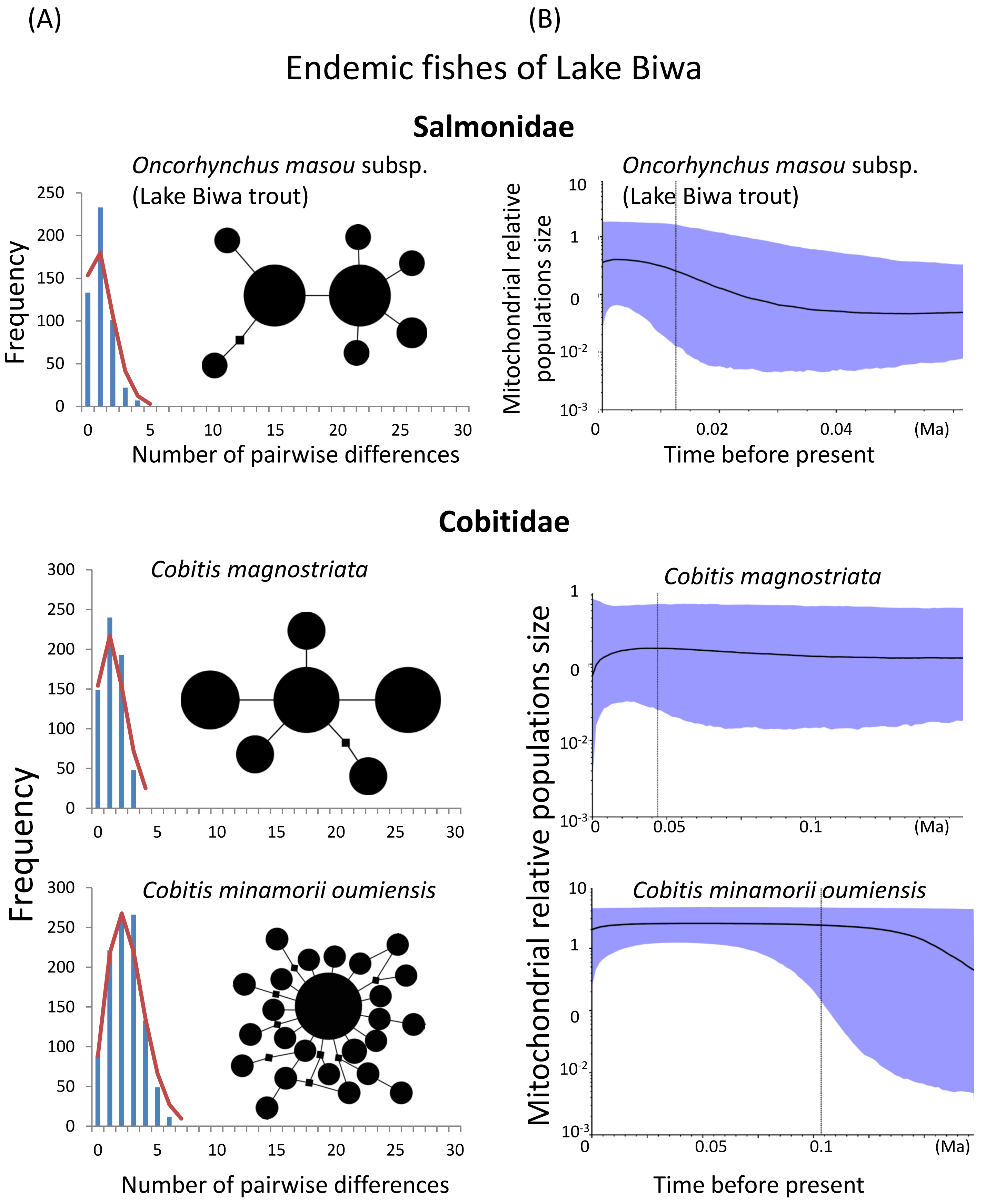
(A)
(B)
Endemic fishes of Lake Biwa
Salmonidae
Oncorhynchus masou subsp. (Lake Biwa trout)
Frequency
Number of pairwise differences
Mitochondrial relative populations size
Time before present
(Ma)
Cobitidae
Cobitis magnostriata
Cobitis minamorii oumiensis
Frequency
Number of pairwise differences
Mitochondrial relative populations size
Time before present

Cyprinidae

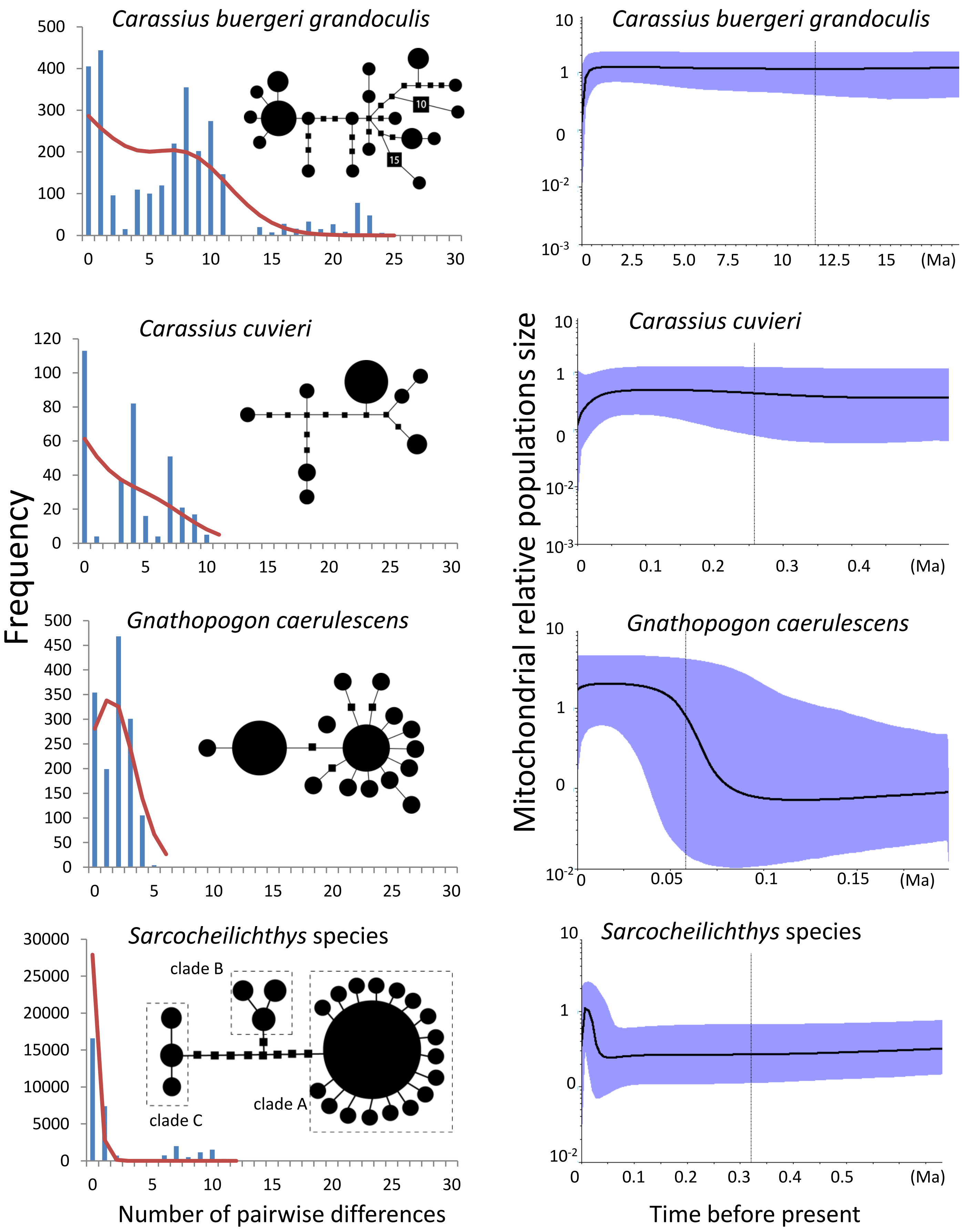

Carassius buergeri grandoculis
Carassius cuvieri
Gnathopogon caerulescens
Sarcocheilichthys species
clade A
clade B
clade C
10
15
Frequency
Number of pairwise differences
Mitochondrial relative populations size
Time before present
(Ma)

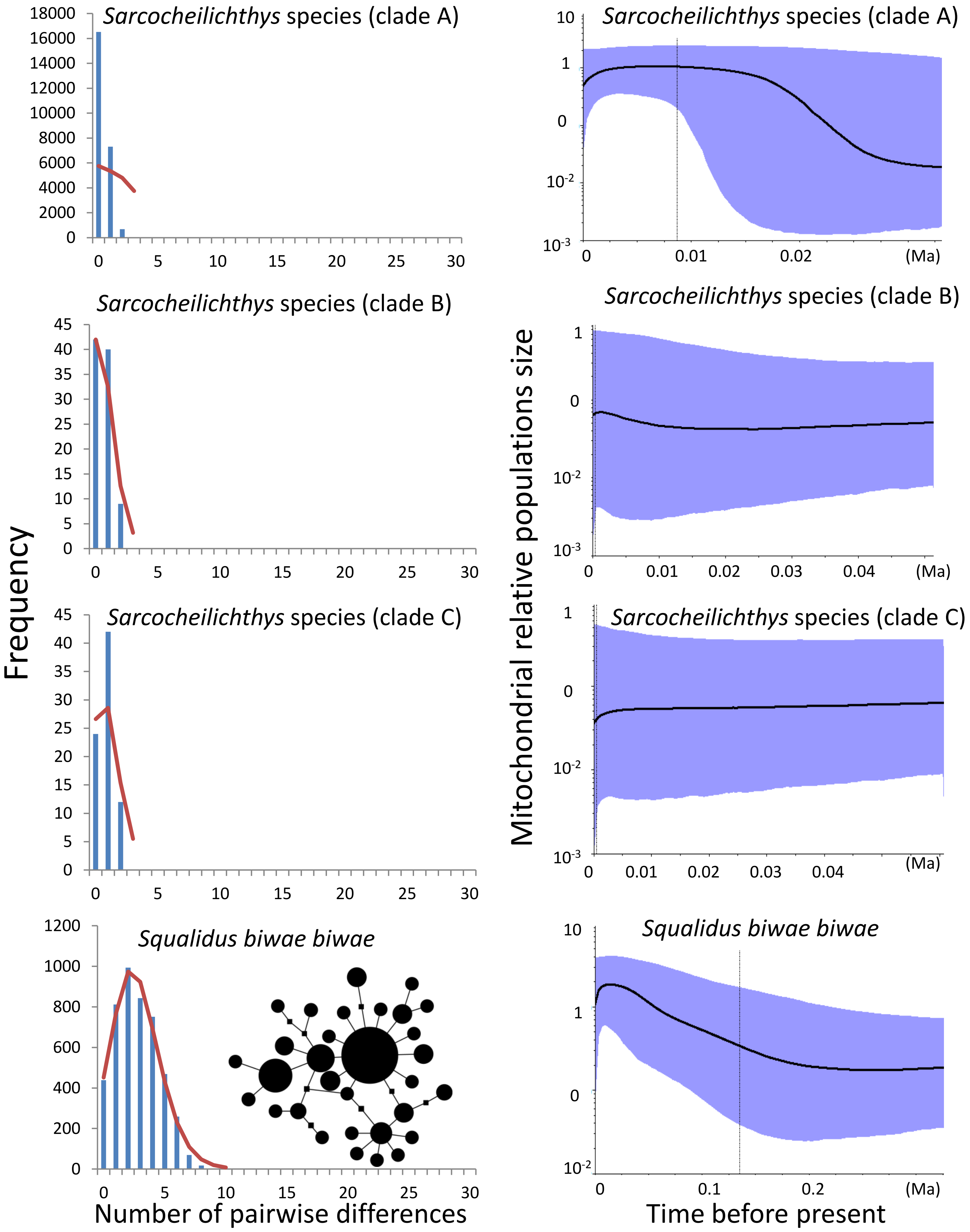

Sarcocheilichthys species (clade A)
Sarcocheilichthys species (clade B)
Sarcocheilichthys species (clade C)
Squalidus biwae biwae
Frequency
Number of pairwise differences
Mitochondrial relative populations size
Time before present
(Ma)

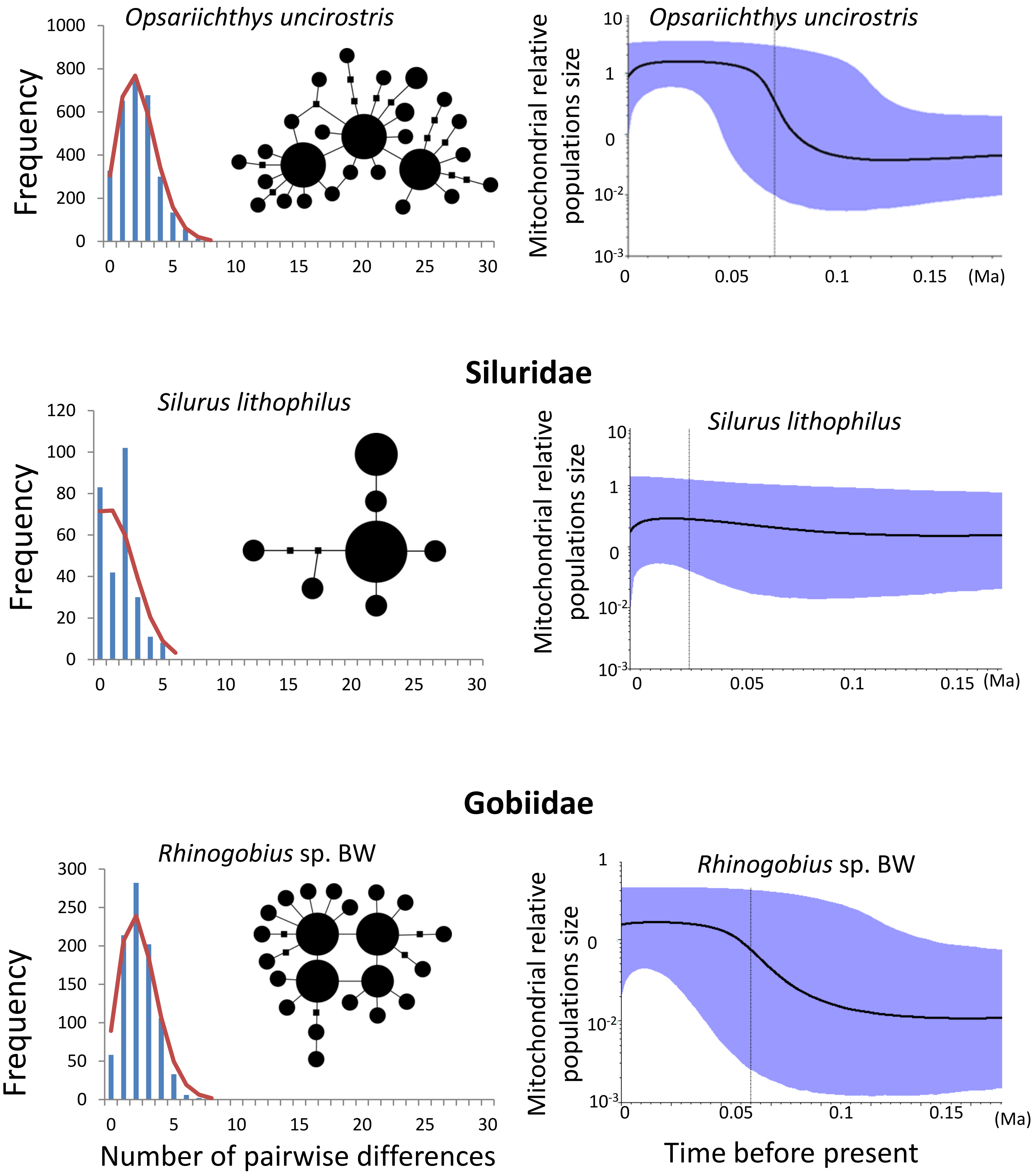
Opsariichthys uncirostris
Frequency
Mitochondrial relative populations size
(Ma)
Siluridae
Silurus lithophilus
Gobiidae
Rhinogobius sp. BW
Number of pairwise differences
Time before present

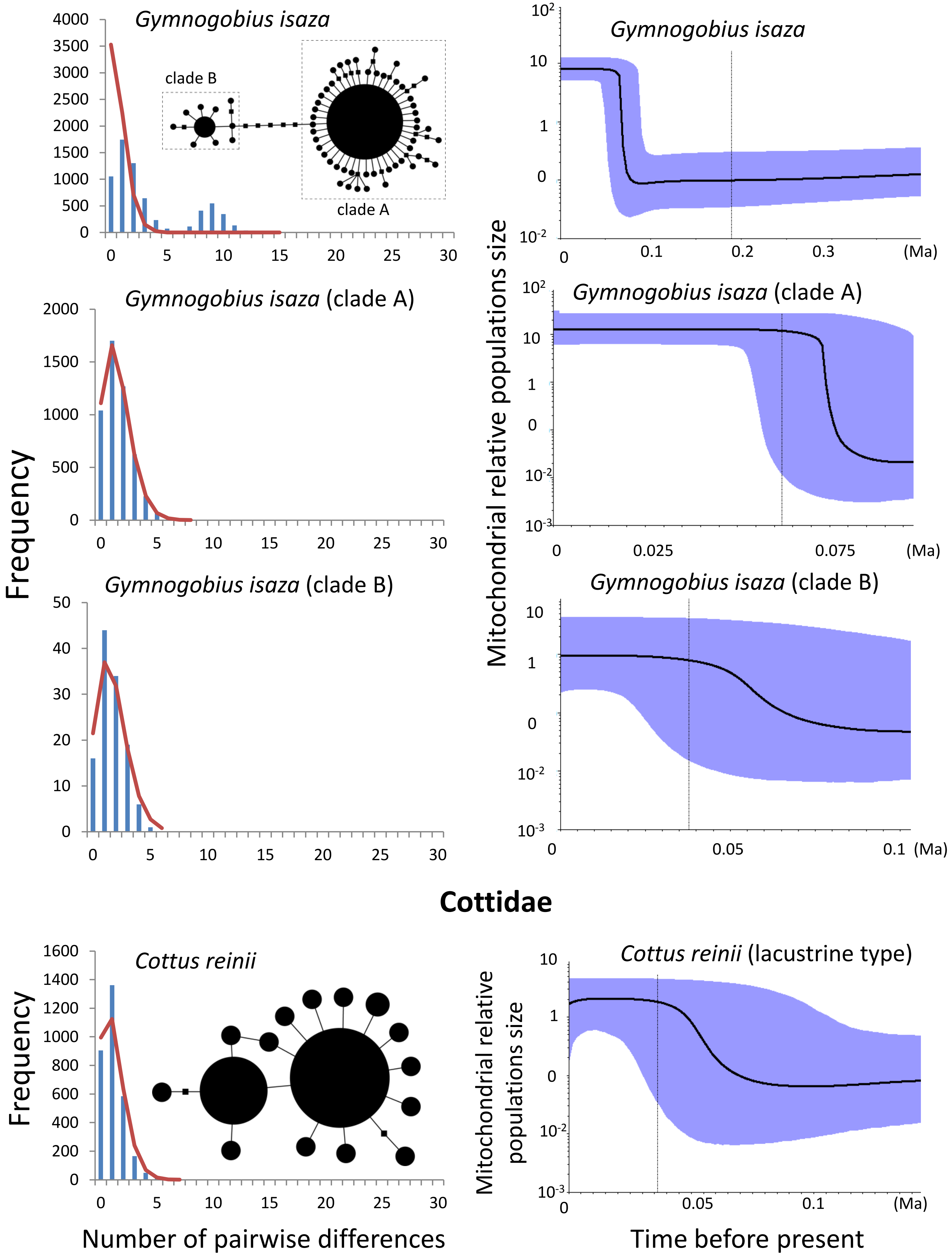

Gymnogobius isaza
clade B
clade A
Gymnogobius isaza (clade A)
Gymnogobius isaza (clade B)
Frequency
Mitochondrial relative populations size
(Ma)
Cottidae
Cottus reinii
Cottus reinii (lacustrine type)
Number of pairwise differences
Time before present

Non-endemic fishes of Lake Biwa

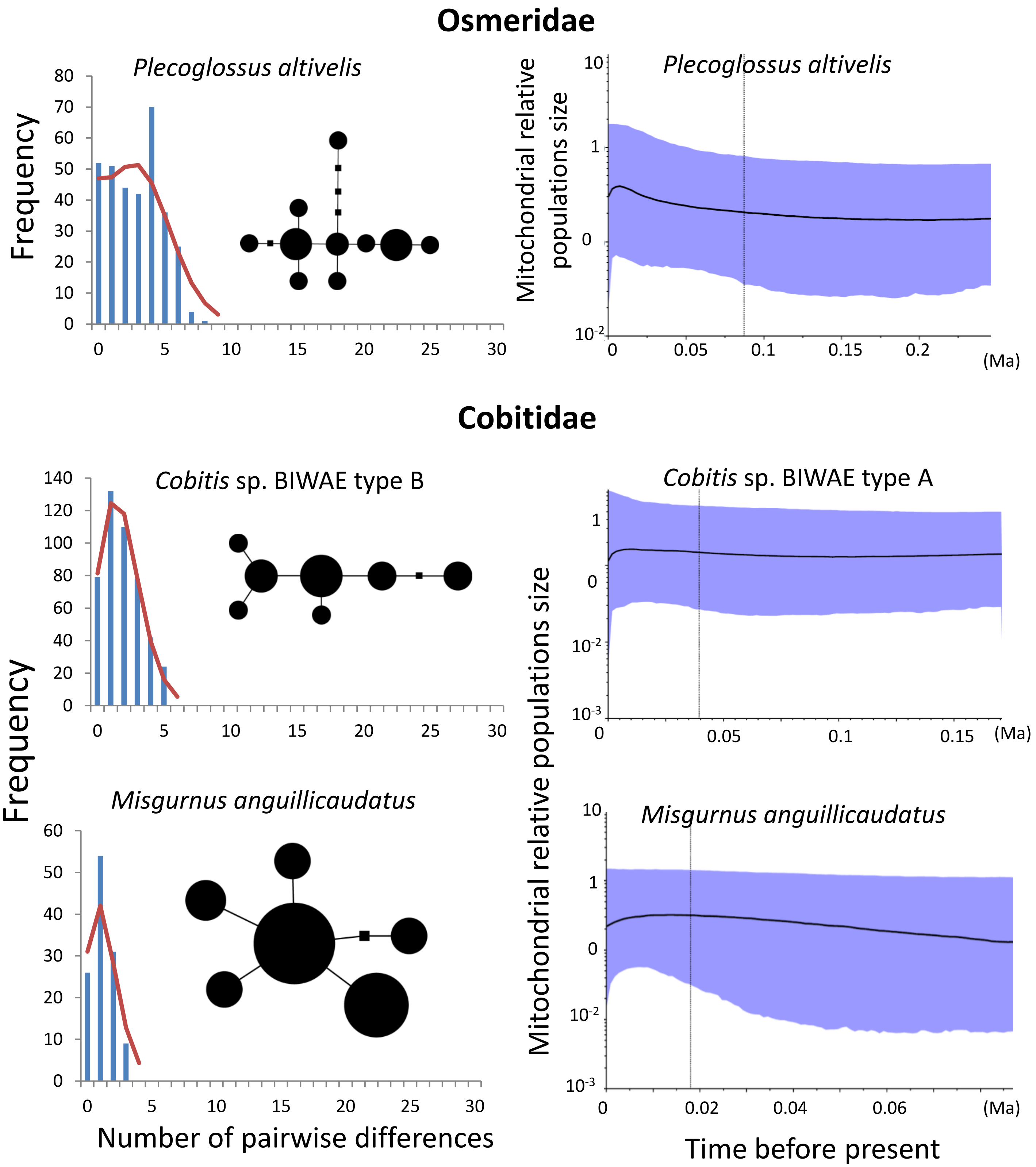

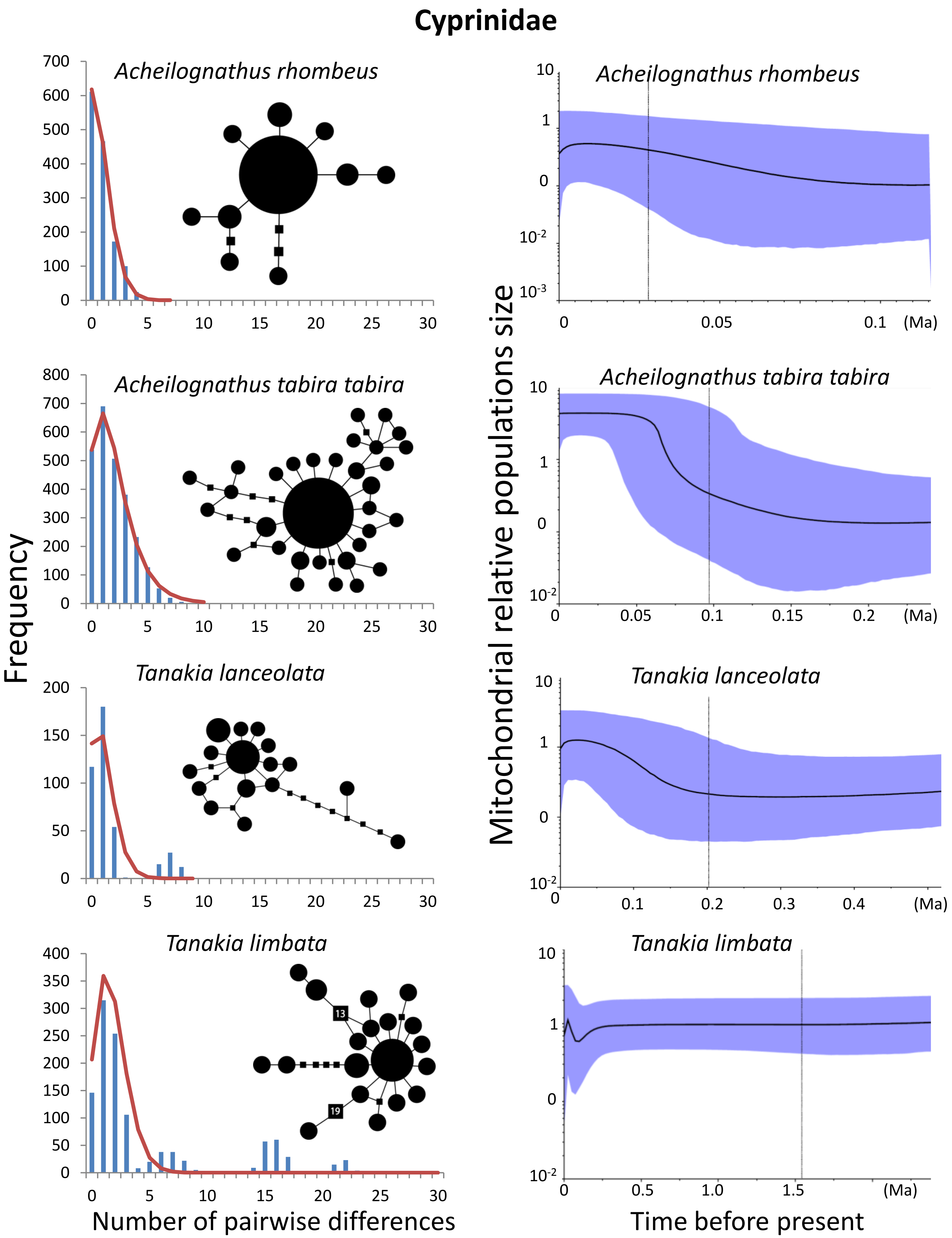
Cyprinidae
Acheilognathus rhombeus
Acheilognathus tabira tabira
Tanakia lanceolata
Tanakia limbata
13
19
Frequency
Number of pairwise differences
Mitochondrial relative populations size
Time before present
(Ma)

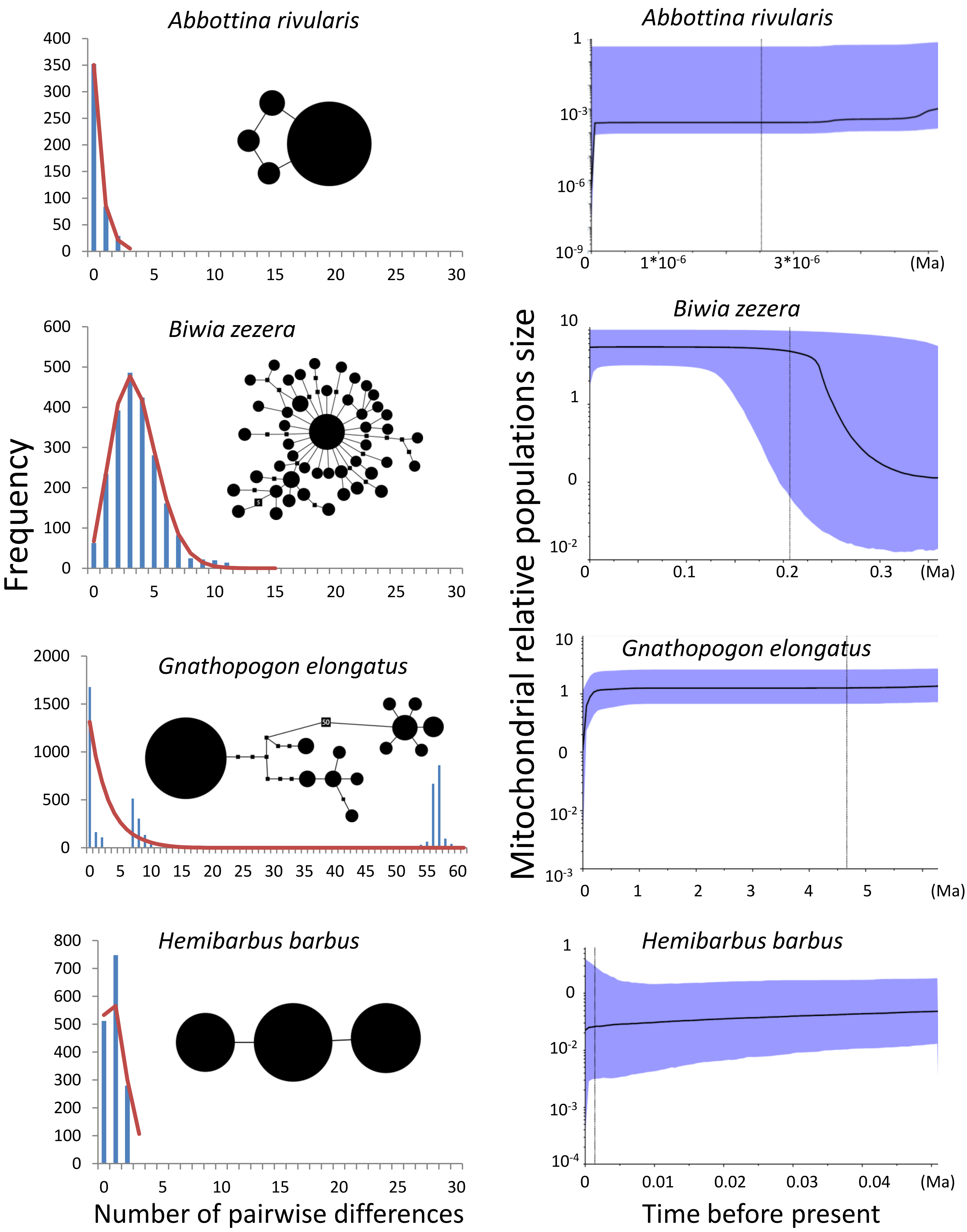

Abbottina rivularis
Biwia zezera
Gnathopogon elongatus
Hemibarbus barbus
Frequency
Number of pairwise differences
Mitochondrial relative populations size
Time before present
(Ma)

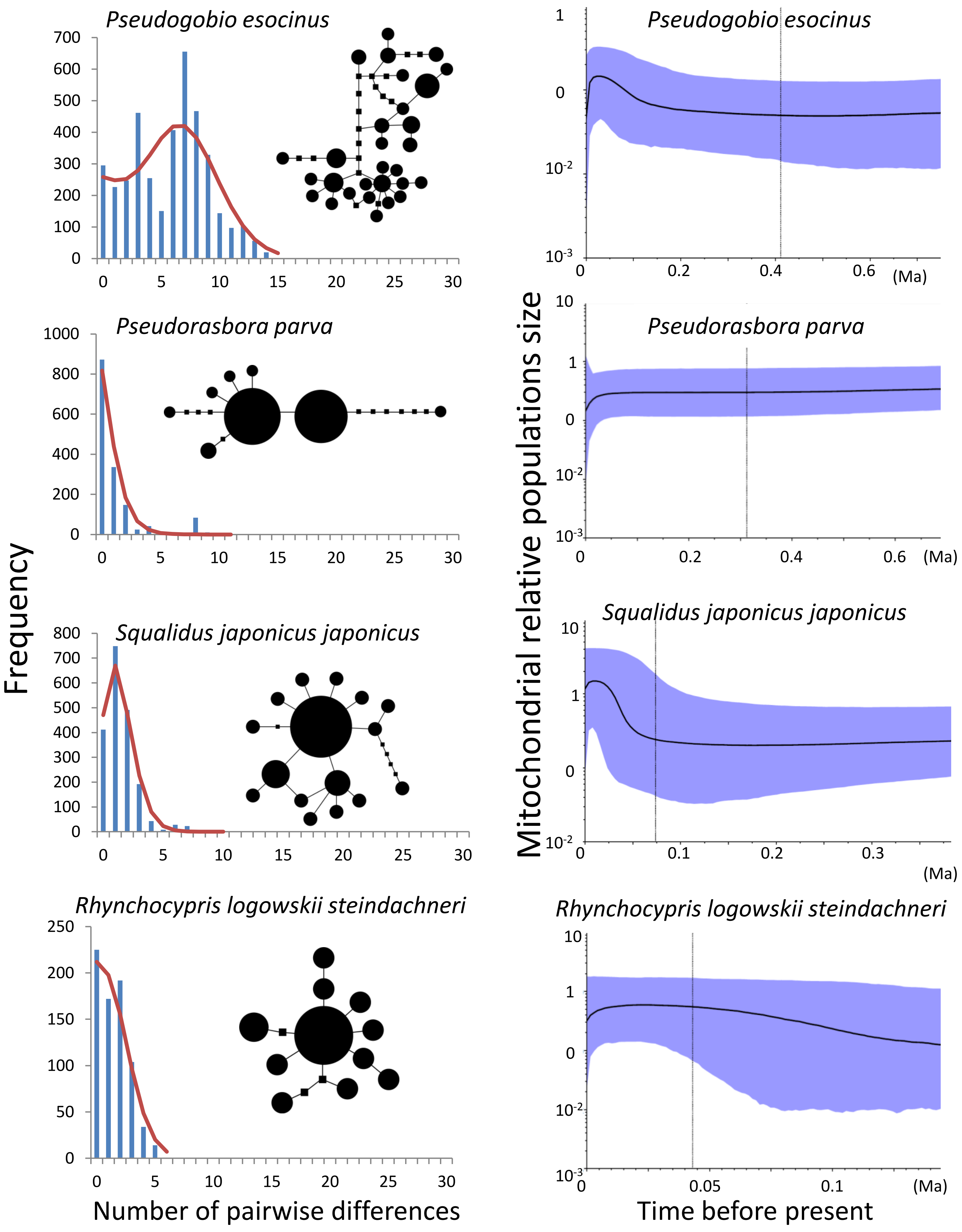

Pseudogobio esocinus
Pseudorasbora parva
Squalidus japonicus japonicus
Rhynchocypris logowskii steindachneri
Frequency
Number of pairwise differences
Mitochondrial relative populations size
Time before present
(Ma)

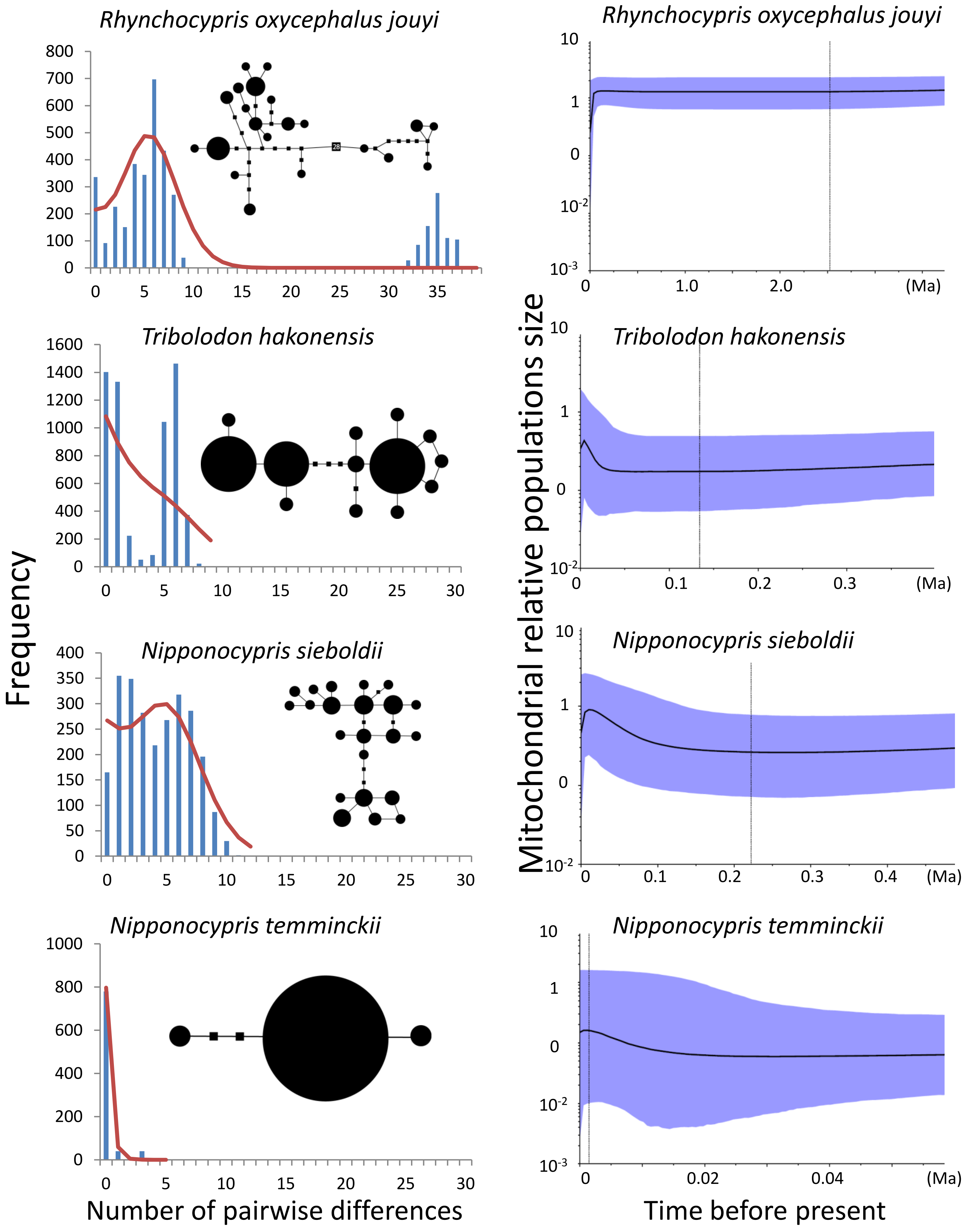

Rhynchocypris oxycephalus jouyi
Tribolodon hakonensis
Nipponocypris sieboldii
Nipponocypris temminckii
Frequency
Number of pairwise differences
Mitochondrial relative populations size
Time before present
(Ma)

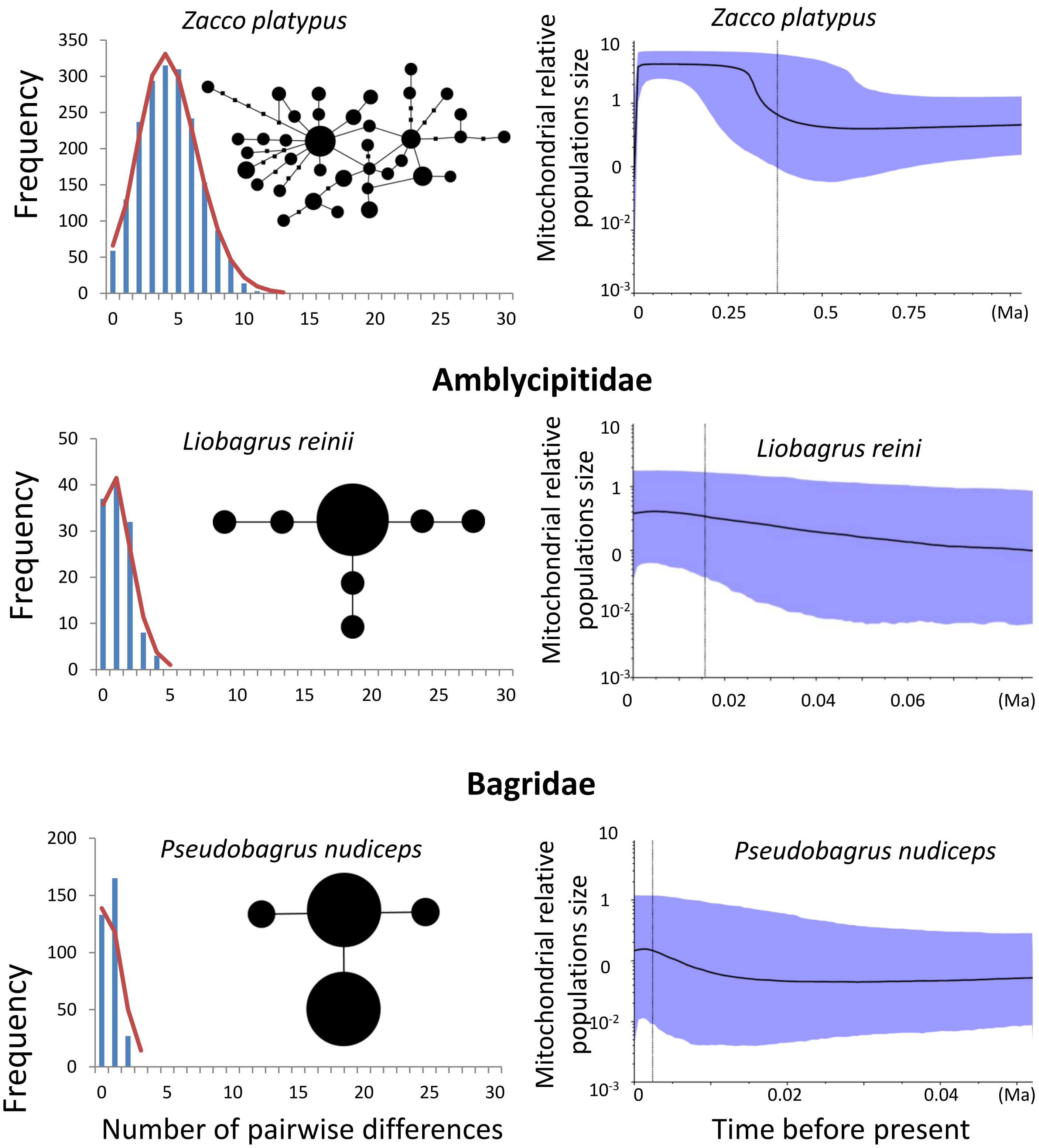

Zacco platypus
Frequency
Mitochondrial relative populations size
Zacco platypus
(Ma)
Amblycipitidae
Liobagrus reinii
Frequency
Mitochondrial relative populations size
Liobagrus reini
(Ma)
Bagridae
Pseudobagrus nudiceps
Frequency
Mitochondrial relative populations size
Pseudobagrus nudiceps
(Ma)
Number of pairwise differences
Time before present

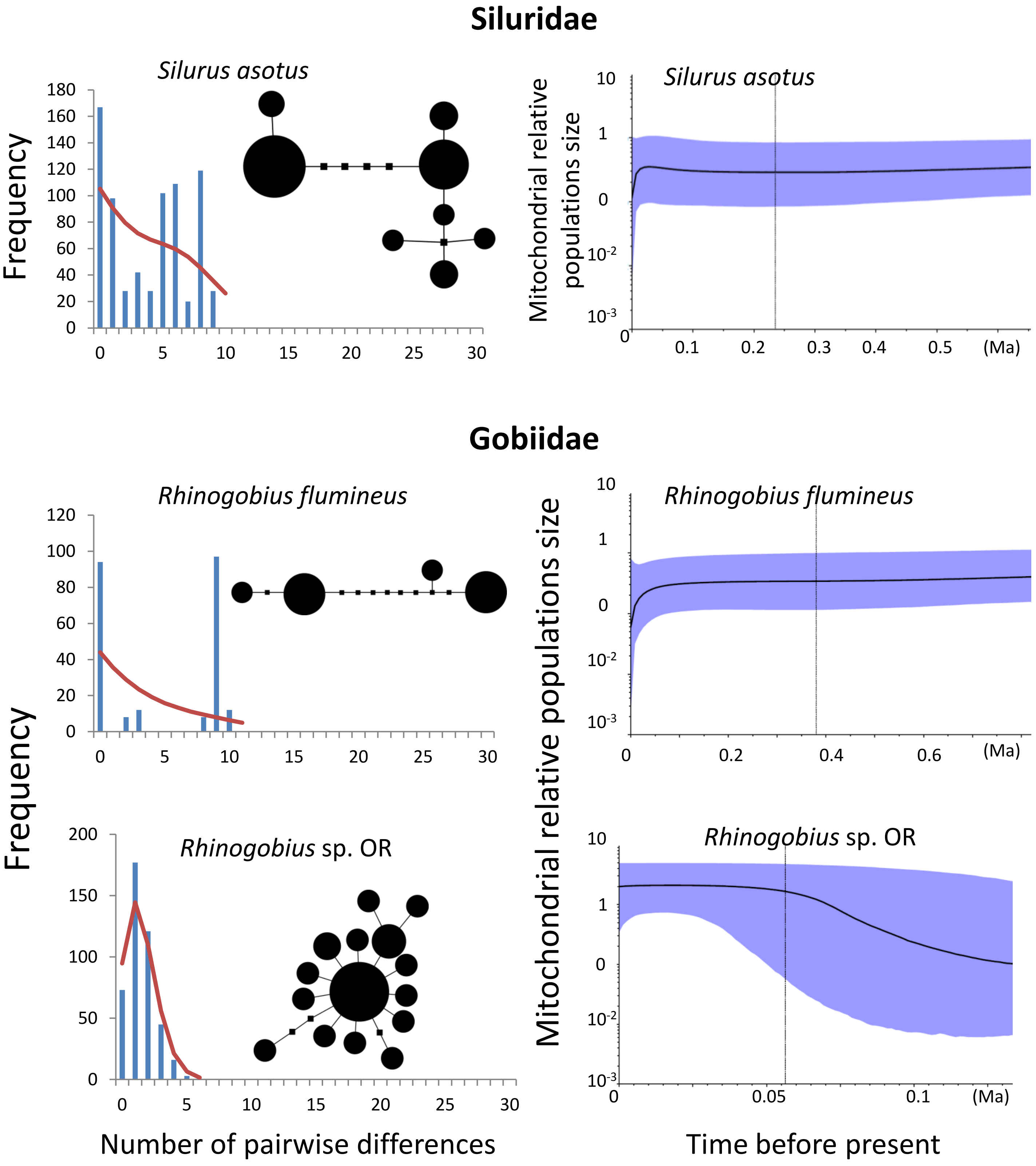
Siluridae
Silurus asotus
Frequency
Silurus asotus
Mitochondrial relative populations size
(Ma)
Gobiidae
Rhinogobius flumineus
Rhinogobius flumineus
Frequency
Mitochondrial relative populations size
(Ma)
Rhinogobius sp. OR
Rhinogobius sp. OR
(Ma)
Number of pairwise differences
Time before present

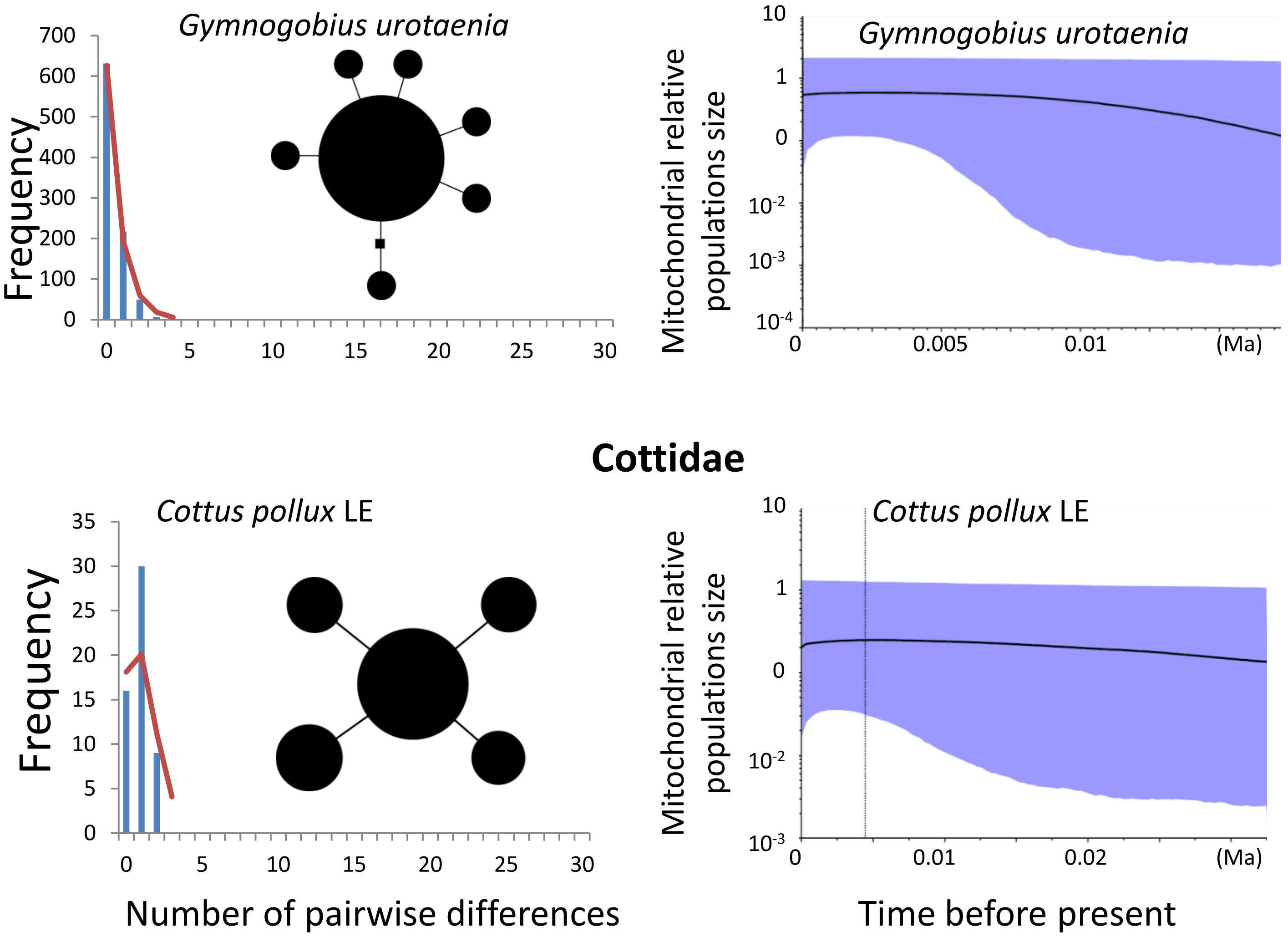
Gymnogobius urotaenia
Frequency
700
600
500
400
300
200
100
0
0
5
10
15
20
25
30
Mitochondrial relative populations size
10
1
0
10-2
10-3
10-4
0.005
0.01
(Ma)
Cottidae
Cottus pollux LE
35
30
25
20
15
10
5
0.02
Number of pairwise differences
Time before present

Fig. S2
