# Supplementary material for: Phylogeny and historical demography of endemic fishes in Lake Biwa: the ancient lake as a promoter of evolution and diversification of freshwater fishes in western Japan
Source: Ecol Evol. 2016 Mar 16;6(8):2601–23. doi: 10.1002/ece3.2070 (PMC4798153; doi:10.1002/ece3.2070)
Supplement: Supplementary file 3 — Figure S3. Statistical parsimony networks of mtDNA cytochrome b haplotypes of selected fishes including Lake Biwa and other local populations. [file ECE3-6-2601-s003.pdf]

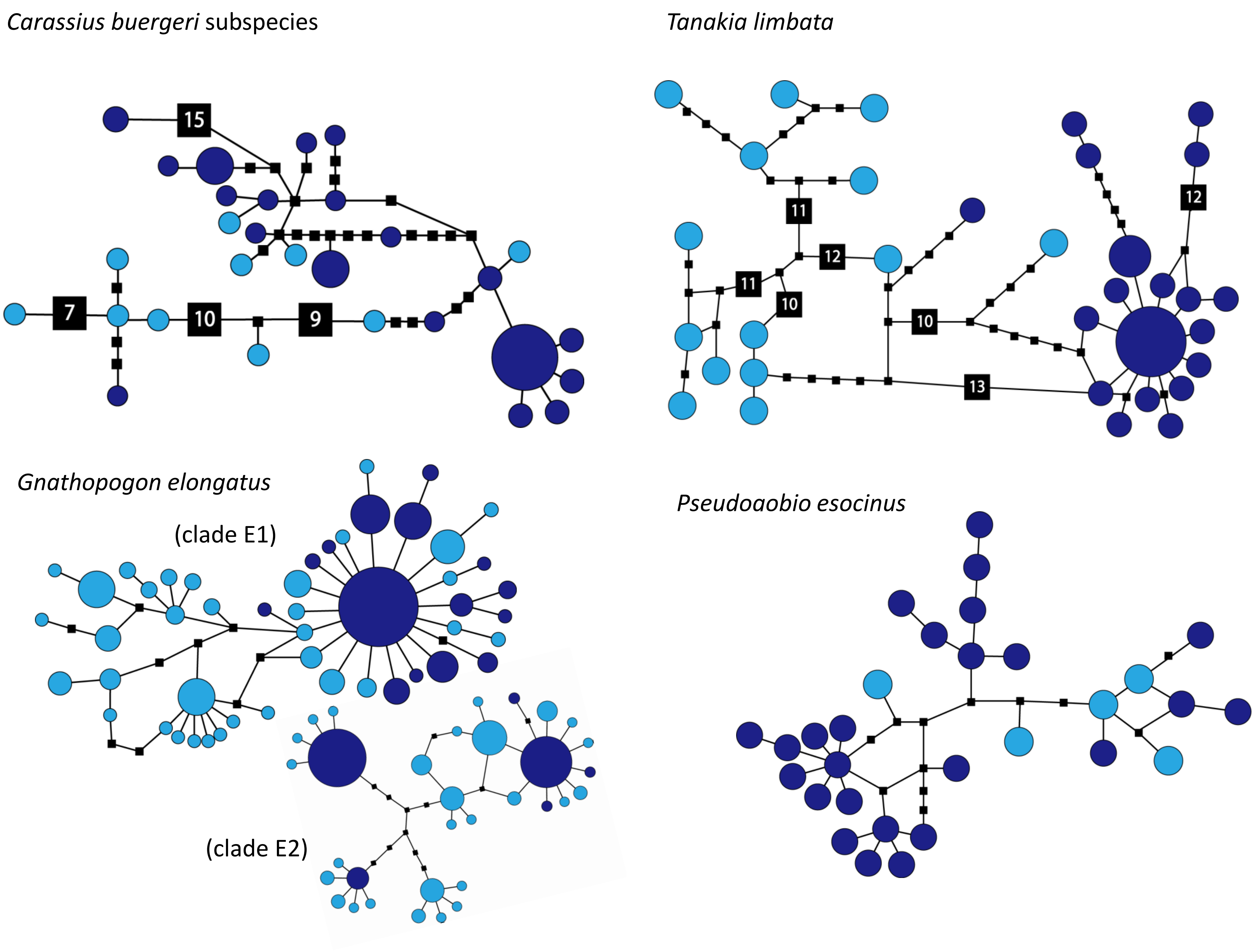
Carassius buergeri subspecies
15
7
10
9
Tanakia limbata
11
12
11
10
10
13
12
Gnathopogon elongatus
(clade E1)
(clade E2)
Pseudogobio esocinus

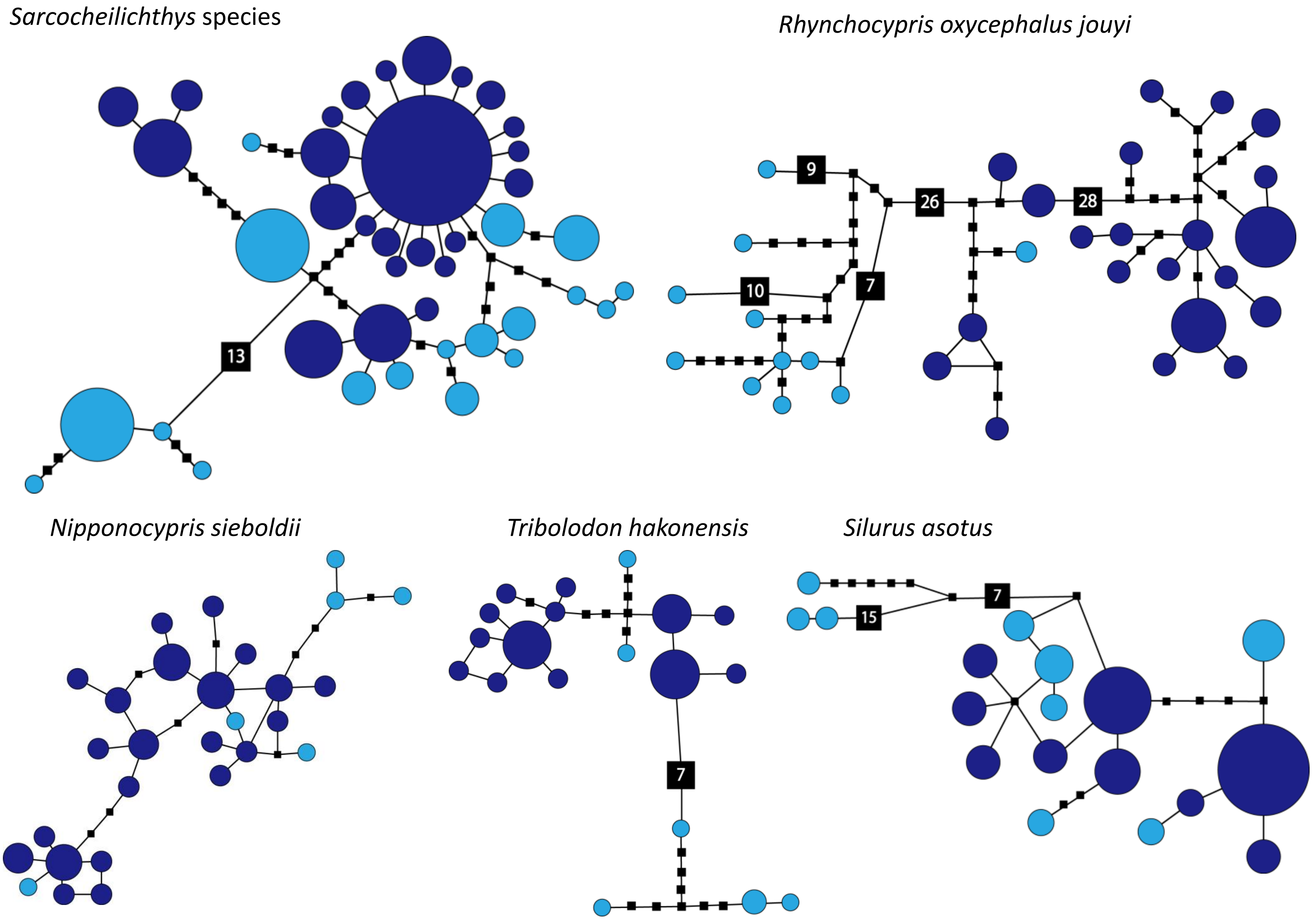
Sarcocheilichthys species
13
Rhynchocypris oxycephalus jouyi
9
26
28
10
7
Nipponocypris sieboldii
Tribolodon hakonensis
7
Silurus asotus
7
15

Fig. S3
