# Supplementary material for: Phylogeny and historical demography of endemic fishes in Lake Biwa: the ancient lake as a promoter of evolution and diversification of freshwater fishes in western Japan
Source: Ecol Evol. 2016 Mar 16;6(8):2601–23. doi: 10.1002/ece3.2070 (PMC4798153; doi:10.1002/ece3.2070)
Supplement: Supplementary file 4 — Figure S4. Distribution maps of the of Japanese freshwater fishes used in this study. The species/subspecies with an asterisk are the closest Lake Biwa endemics. [file ECE3-6-2601-s004.pdf]

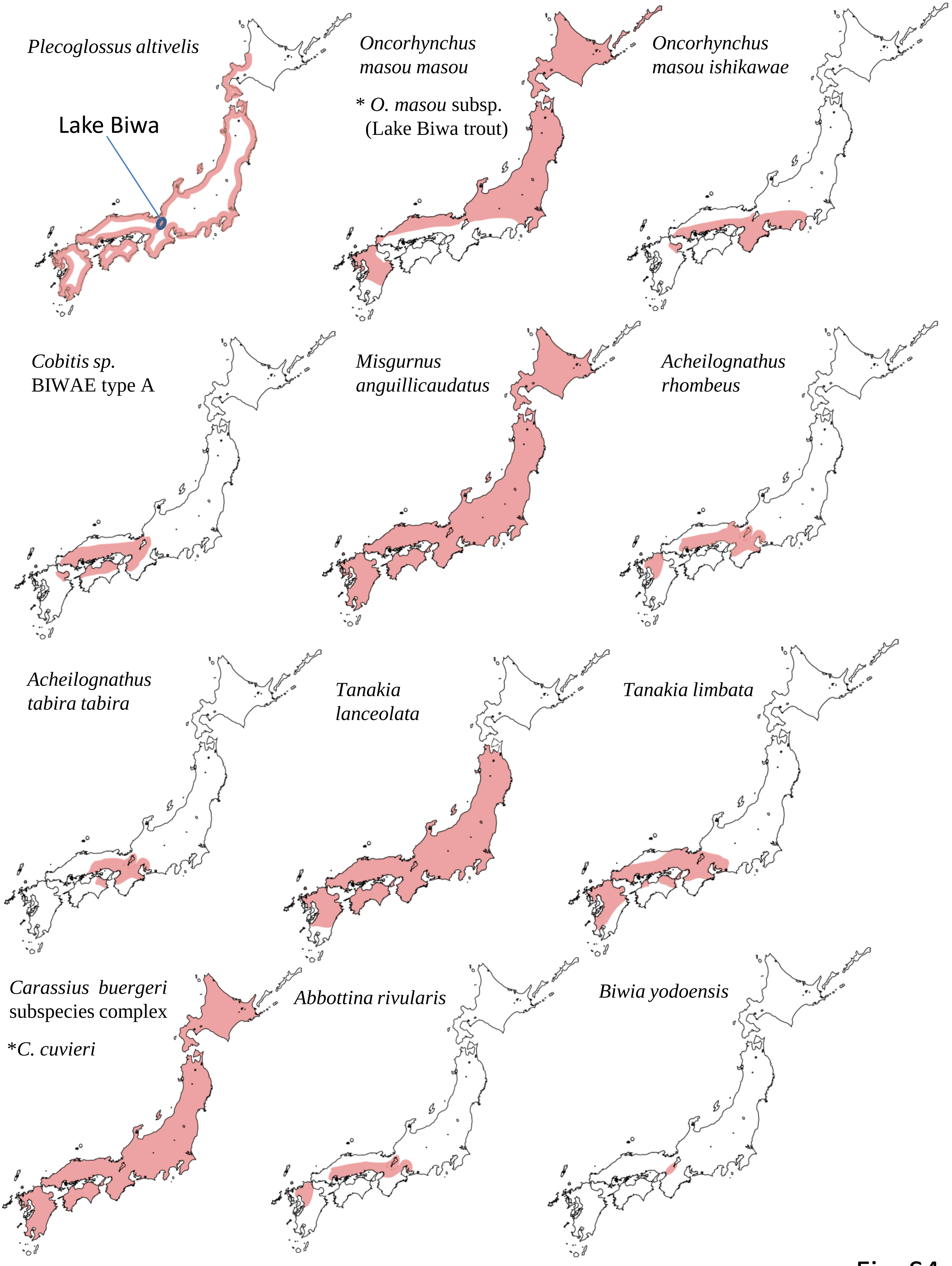

Plecoglossus altivelis
Lake Biwa
Oncorhynchus masou masou
* O. masou subsp. (Lake Biwa trout)
Oncorhynchus masou ishikawae
Cobitis sp. BIWAE type A
Misgurnus anguillicaudatus
Acheilognathus rhombeus
Acheilognathus tabira tabira
Tanakia lanceolata
Tanakia limbata
Carassius buergeri subspecies complex
*C. cuvieri
Abbottina rivularis
Biwia yodoensis

Fig. S4

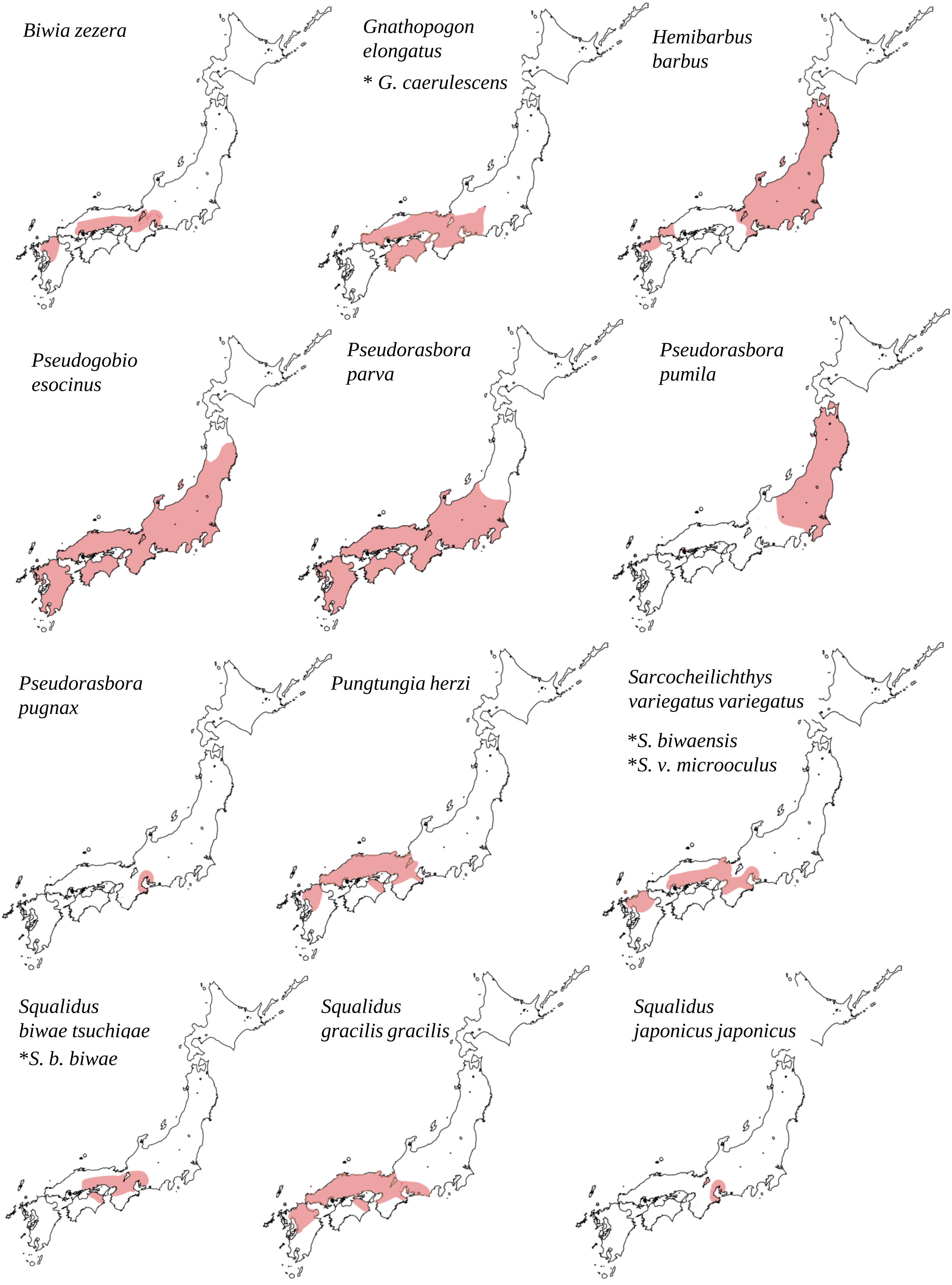
Biwia zezera
Gnathopogon elongatus
* G. caerulescens
Hemibarbus barbus
Pseudogobio esocinus
Pseudorasbora parva
Pseudorasbora pumila
Pseudorasbora pugnax
Pungtungia herzi
Sarcocheilichthys variegatus variegatus
*S. biwaensis
*S. v. microoculus
Squalidus biwae tsuchigae
*S. b. biwae
Squalidus gracilis gracilis
Squalidus japonicus japonicus

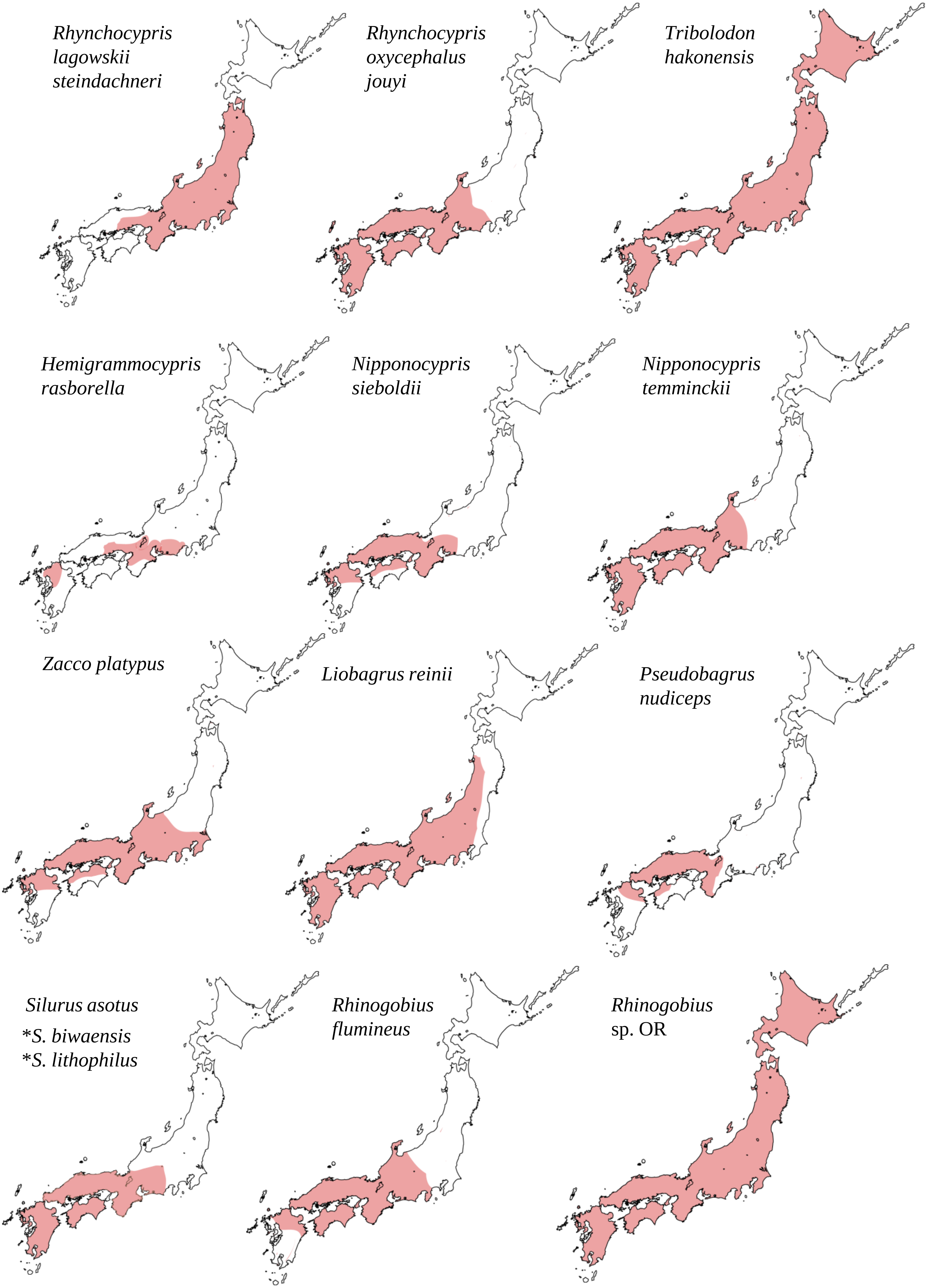
Rhynchocypris lagowskii steindachneri
Rhynchocypris oxycephalus jouyi
Tribolodon hakonensis
Hemigrammocypris rasborella
Nipponocypris sieboldii
Nipponocypris temminckii
Zacco platypus
Liobagrus reinii
Pseudobagrus nudiceps
Silurus asotus
**S. biwaensis*
**S. lithophilus*
Rhinogobius flumineus
Rhinogobius sp. OR

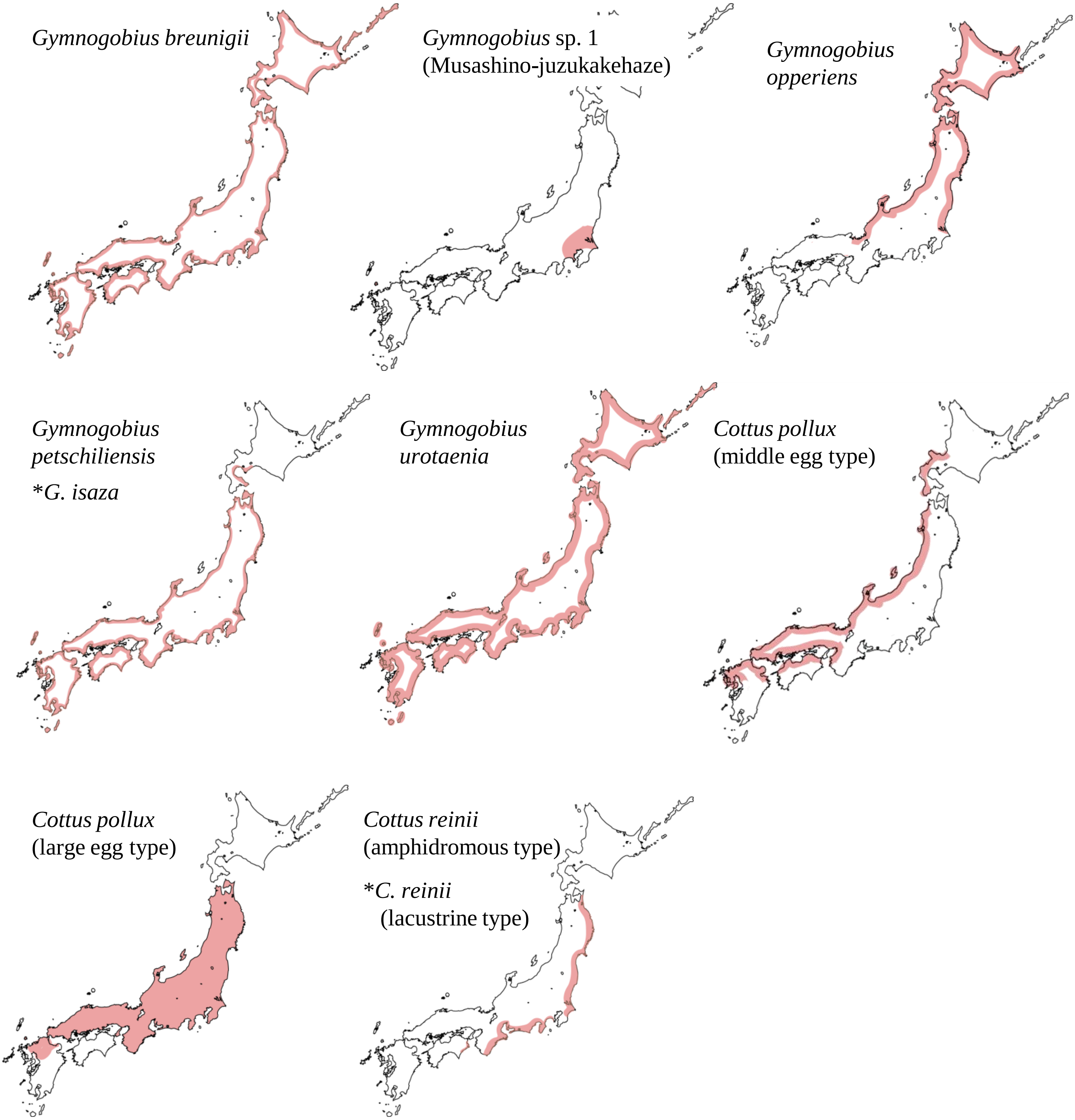
Gymnogobius breunigii
Gymnogobius sp. 1 (Musashino-juzukakehaze)
Gymnogobius opperiens
Gymnogobius petschiliensis
*G. isaza
Gymnogobius urotaenia
Cottus pollux (middle egg type)
Cottus pollux (large egg type)
Cottus reinii (amphidromous type)
*C. reinii (lacustrine type)
