# Supplementary material for: Phylogeny and historical demography of endemic fishes in Lake Biwa: the ancient lake as a promoter of evolution and diversification of freshwater fishes in western Japan
Source: Ecol Evol. 2016 Mar 16;6(8):2601–23. doi: 10.1002/ece3.2070 (PMC4798153; doi:10.1002/ece3.2070)
Supplement: Supplementary file 6 [file ECE3-6-2601-s006.docx]

**Supporting Information**

**References (Table S4)**

Aoyama, J., M. Nishida, and K.Tsukamoto. 2001. Molecular phylogeny and evolution of the freshwater eel, genus *Anguilla*. Mol. Phylogenet. Evol. 20:450–459.

Aoyama, J., S. Watanabe, S. Ishikawa, M. Nishida, and K. Tsukamoto. 2000. Are morphological characters distinctive enough to discriminate between two species of freshwater eels, *Anguilla celebesensis* and *A. interioris*? Ichthyol. Res. 47:157–161.

Ducroz, J. F., V. Volobouev, and L. Granjon. 2001. An assessment of the systematics of arvicanthine rodents using mitochondrial DNA sequences: evolutionary and biogeographical implications. J. Mamm. Evol. 8:173–206.

Iguchi, K. I., Y. Tanimura, and M. Nishida. 1997. Sequence divergence in the mtDNA control region of amphidromous and landlocked forms of ayu. Fish. Sci. 63:901–905.

Inoue, J. G., M. Miya, K. Tsukamoto, and M. Nishida. 2000. Complete mitochondrial DNA sequence of the Japanese sardine *Sardinops melanostictus*. Fish. Sci. 66:924–932.

Miya, M., and M. Nishida. 1996. Molecular phylogenetic perspective on the evolution of the deep-sea fish genus *Cyclothone* (Stomiiformes: Gonostomatidae). Ichthyol. Res. 43:375–398.

Miya, M., and M. Nishida. 2000. Use of mitogenomic information in teleostean molecular phylogenetics: a tree-based exploration under the maximum-parsimony optimality criterion. Mol. Phylogenet. Evol. 17:437–455.

Palumbi, S., A. Martin, S. Romano, W. O. McMillam, L. Stice, and G. Grabowski. 1991. The Simple Tools Guide to PCR*.* Department of Zoology and Kewalo Marine Laboratory, University of Hawaii Honololulu.

Ward, R. D., T. S. Zemlak, B. H. Innes, P. R., Last, and P. D. Hebert. 2005. DNA barcoding Australia's fish species. Phil. Trans. R. Soc. B 360:1847–1857.

Yokoyama, R., and A. Goto. 2002. Phylogeography of a freshwater sculpin, *Cottus nozawae*, from the northeastern part of Honshu Island, Japan. Ichthyol. Res. 49:147–155.

**Figure regends**

**Fig. S1.** Bayesian phylogenetic trees of each fish group, including outgroup species, based on mtDNA 16S, CO1, ND5 cyt *b*, and CR gene sequences. The trees were dated with the random local clock model with node age constraints (C1–C6; see Table 2). All nodes are supported by a Bayesian posterior probability of 1.0, except those denoted by *numbers*. *Horizontal bars* *at nodes* show credible intervals as 95% highest posterior density. For codes of nodes (a–o), see Table 3.

**Fig. S2.** **(A)** Mismatch distributions and statistical parsimony networks of mtDNA cytochrome *b* haplotypes of each species. **(B)** The results of Bayesian skyline plots for fishes in Lake Biwa.

**Fig. S3.** Statistical parsimony networks of mtDNA cytochrome *b* haplotypes of selected fishes including Lake Biwa and other local populations. The *sizes of the circles* are proportional to haplotype frequencies. The *dark circles* represent haplotypes found in Lake Biwa and rivers connected to the lake. The *pale circles* represent haplotypes detected from the areas outside Lake Biwa.

**Fig. S4.** Distribution maps of the of Japanese freshwater fishes used in this study. The species/subspecies with an asterisk are the closest Lake Biwa endemics.
